# Quantitative trait loci for agronomic traits in tetraploid wheat for enhancing grain yield in Kazakhstan environments

**DOI:** 10.1371/journal.pone.0234863

**Published:** 2020-06-23

**Authors:** Shynar Anuarbek, Saule Abugalieva, Nicola Pecchioni, Giovanni Laidò, Marco Maccaferri, Roberto Tuberosa, Yerlan Turuspekov

**Affiliations:** 1 Institute of Plant Biology and Biotechnology, Almaty, Kazakhstan; 2 al-Farabi Kazakh National University, Almaty, Kazakhstan; 3 Kazakh National Agrarian University, Almaty, Kazakhstan; 4 Research Centre for Cereal and Industrial Crops, CREA, Foggia, Italy; 5 Department of Agricultural and Food Sciences, University of Bologna, Bologna, Italy; University of Helsinki, FINLAND

## Abstract

Durum wheat (*Triticum turgidum* L. ssp. *durum*) is one of the top crops in Kazakhstan, where it is cultivated in different ecological niches, mainly at higher latitudes in the steppe zone of the northern region. Therefore, local breeding programs for durum wheat are primarily focused on selection for high productivity in Northern Kazakhstan based on the introduction of promising foreign germplasm and the adoption of marker-assisted selection. In this study, a world tetraploid wheat collection consisted of 184 primitive and domesticated accessions, which were previously genotyped using 16,425 polymorphic SNP markers, was field-tested in Northern and South-eastern Kazakhstan. The field tests have allowed the identification of 80 durum wheat promising lines in Northern Kazakhstan in comparison with a local standard cultivar. Also, GGE (Genotype and Genotype by Environment) biplot analyses for yield performance revealed that accessions of *T*. *dicoccum*, *T*. *carthlicum*, and *T*. *turanicum* also have potential to improve durum wheat yield in the region. The genome-wide association study (GWAS) has allowed the identification of 83 MTAs (marker-trait associations) for heading date, seed maturation time, plant height, spike length, number of fertile spikes, number of kernels per spike, and thousand kernel weight. The comparison of the 83 identified MTAs with those previously reported in GWAS for durum wheat suggests that 38 MTAs are presumably novel, while the co-localization of a large number of MTAs with those previously published confirms the validity of the results of this study. The MTAs reported herewith will provide the opportunity to implement marker-assisted selection in ongoing durum wheat breeding projects targeting higher productivity in the region.

## Introduction

Durum wheat (2*n* = 28, AABB, *Triticum turgidum* L. ssp. *durum*) is an increasingly important worldwide commodity driven by the success of the Mediterranean diet and cultivated in around 18 million hectares worldwide [1]. In 2019, a total of 465 thousand ha of durum wheat were planted in Kazakhstan [2] with an annual production of up to 500 thousand tons of grain and exports up to 385 thousand tons, mainly to Italy (51%), Russia (25%), Turkey (8%) and Tunisia (5%) [3].

Worldwide, durum wheat is cultivated in areas with low seasonal rainfall and frequently affected by drought, the main limiting factor for grain yield [4, 5]. Studies of yield performance in different ecological niches are important for optimizing breeding activities across wheat-growing regions. During the last decades, breeders have developed varieties that are highly productive and widely adapted to contrasting environments [6, 7]. In Kazakhstan, wheat is prevalently cultivated at higher latitudes in the steppe zone of the northern region [8]. Breeding programs for durum wheat are primarily focused on increased yield potential and early maturation to optimize grain yield and quality in the conditions of Nothern Kazakhstan [9]. Hence, producing a high yield will depend on the knowledge of both environmental and genetic yield-limiting factors [10].

Wheat yield and related traits are controlled by multiple quantitative trait loci (QTLs), each contributing to a significant, although a small fraction of phenotypic variance, which generally interacts with environmental factors (QTL x E), and/or epistatically [11]. Therefore, a detailed understanding of the genetic make-up of quantitative traits will be instrumental to deploy marker-assisted selection to enhance selection gain of traditional breeding programs.

The evaluation of genetically diverse genetic materials well adapted and commercially suitable for specific end-use products, is an essential prerequisite to identify beneficial allelic variation to enhance durum wheat productivity while preserving its quality. Therefore, the evaluation of a genetically diverse collection of tetraploid wheat accessions plays a pivotal role for the dissection of the QTLome controlling the relevant agronomic traits [11]. Previously, a wild and domesticated wheat collection was evaluated to identify QTLs for yield components and grain quality [12, 13], and for resistance to *Puccinia graminis* f. sp. *tritici* (*Pgt*) [14]. Multiple environment trials, especially for grain yield traits, are commonly used to assess the performance of genotypes across a range of locations and years [15]. Accordingly, the stable expression of a QTL across a broad range of agrometeorological conditions is a critical factor when breeding for wide adaptation and yield stability. As costs for high-hroughput genotyping are decreasing, genome-wide association studies (GWAS) are increasingly adopted for the detection of QTLs associated with durum wheat agronomic traits, with the final goal of accelerating local breeding activities based on the application of marker-assisted selection [16, 17]. Currently, GWAS has been successfully used in durum wheat for identification of QTLs for yield components in Europe [18], Central America [19], South America [20], Africa [21, 22], Asia [23], and in multilocation studies [16, 17]. Although GWAS has proven to be very effective for capturing relevant marker-trait associations (MTAs) for yield components, results reported from studies in different regions of the world are revealing the tendency for a strong influence of the environmental conditions with significant genotype x environment interaction (GEI) revealed. For example, GWAS results obtained from multilocation studies related to the identification of QTLs for yield performance showed different responses, and QTLs for yield components were identified in different parts of the genome [16, 17], similarly to what reported in common wheat [24, 8] and barley [25, 26, 27]. Therefore, the success of regional projects largely relies on local GWA studies based on the evaluation of genetically diverse wild and domesticated germplasm. The main goal of this work was to identify MTAs for agronomic traits and yield in durum wheat based on GWAS of the world tetraploid wheat collection (TWC) evaluated in field trials conducted in two contrasting environments in Kazakhstan. Ultimately, the results presented herewith will contribute to the implementation of marker-assisted selection of durum wheat in this Central Asian country.

## Materials and methods

### Plant materials and field trials

The tetraploid wheat collection (TWC) used in this study was initially comprised of 225 accessions of worldwide origin that were selected to represent the phenotypic variability for the grain yield component traits that were evaluated in this study. The seeds were provided by the Research Centre for Cereal and Industrial Crops (Foggia, Italy) and the Department of Agricultural and Food Sciences of the University of Bologna (Bologna, Italy). The panel included seven accessions of wild emmer wheat (*Triticum turgidum* ssp. *dicoccoides*) and 177 accessions of six cultivated sub-species of *Triticum turgidum* (namely: ssp. *durum*, *turanicum*, *polonicum*, *turgidum*, *carthlicum*, and *dicoccum*). The name and place of origin for each accession are listed in (S1 Table). The details on the genetic diversity, population structure and LD patterns of this collection of tetraploid wheats were previously described in [28] and [13]. The 41 accessions that did not reach the seed maturation stage due to vernalization and/or photoperiod requirements were not considered for GWAS analysis.

The 184 accessions of the TWC were grown in the North Kazakhstan Agricultural Experimental Station in 2018 and 2019, hereafter reported as NK18 and NK19, as well as in South-East Kazakhstan in the experimental fields of the Kazakh Research Institute of Agriculture and Plant Industry, hereafter reported as SEK18 and SEK19. The two locations represent contrasting growing areas for altitude, elevation above sea level, and soil quality. S2 Table reports the details for their locations, meteorological parameters, and soil conditions.

Field experiments were conducted according to a randomized complete block design with two replications. Each accession was planted in two rows, 25 seeds per row, with a row spacing of 15 cm, with no irrigation during the growing season, hence under rainfed conditions.

### Phenotyping

The tetraploid wheat panel was evaluated for days to heading (HD, days), seed maturation time (SMT, days), plant height (PH, cm), spike length (SL, cm), number of fertile spikes (NFS), number of kernels per spike (NKS), thousand kernel weight (TKW, g) and yield per plant (YPP, g). Previously, this set was evaluated for yield components in the field, and results were reported in [29]. Days to heading (HD) was recorded as the number of days from emergence to the day when half of the spikes appeared in 50% of the plants. Seed maturation time (SMT) was measured as number of days between heading time and maturation time. After harvesting, PH, SL, NFS, NKS, TKW, YPP were measured as the mean of five plants for each accession. Plant height (PH) was measured at harvest maturity, when the maximum height was achieved, from the ground level to the top of the spikes (excluding awns). Spike length (SL) was determined by measuring the spikes from the base of the first spikelet to the tip of the most terminal spikelet, excluding the awns. NFS and NKS were measured by counting the number of fertile spikes and kernels of five plants, respectively. TKW was determined by weighing 100 seeds and multiplying the number by 10. Pearson correlation coefficients (*r*) were calculated among traits using STATISTICA 13.2 (Statistica, Statsoft Inc., Tulsa, OK, USA). GGE Biplot methods were employed by using the GenStat package (17th release, VSN International, Hertfordshire, UK). The principal coordinate analysis (PCoA) was performed for the relationship analyses of species with different origins based on pairwise population PhiPT values using GenAlEx 6.5 [30, 31].

### SNP genotyping

The genotyping data for 16,425 SNP markers (Illumina® iSelect 90K wheat SNP assay, TraitGenetics GmbH, Gatersleben, Germany) was provided by Nicola Pecchioni and Giovanni Laidò (Research Centre for Cereal and Industrial Crops, Foggia, Italy). The genotypic data was already filtered from markers with >10% missing data and with <0.1 minor allele frequency (MAF). The details of SNP genotyping and dataset filtering were described in [32, 14].

### Association mapping analysis

Association mapping analysis was performed using TASSEL 5.2.38 software (http://www.maizegenetics.net/tassel) and GAPIT R package [33]. Marker-trait associations (MTAs) between SNP markers and agronomic traits were detected by using the mixed linear model (MLM) that based on the kinship matrix (K) and the population structure matrix (Q) [34]. The genetic structure of the collection (Q matrix) was determined with Bayesian methods using the STRUCTURE software [35], with the optimum number of subpopulations (K) equal to three for the whole collection and six for the durum subgroup, as described in [14]. In the current study, each year-location combination was considered as a separate environment.

In agreement with the linkage disequilibrium (LD) estimates determined by [14], the value of *r*^*2*^ = 0.3 was used as the confidence interval to declare significant SNPs associated with the examined yield component traits. The significant associations were selected after the application of a threshold bar at *P* < 1.96E^-4^. The Quantile-Quantile (Q-Q) plots were analyzed to confirm the correction due to both K and Q matrices usage. All the significant MTAs located on the durum wheat consensus map within short map intervals (10 cM) were grouped into a single QTL. Only those QTLs confirmed in more than one environment, or the two groups (whole collection, durum sub-group) were considered as a stable association with the trait evaluated.

The consensus map of tetraploid wheat described by [36] was used to assign a genomic location of SNP markers associated with QTL for each trait. Graphical representation of the genetic position of MTAs was carried out using MapChart 2.2 software [37].

The genetic position of the identified QTLs was compared with data obtained and published in other studies in tetraploid wheat for the same traits. The sequences of the SNP tagged markers within the estimated interval of each QTL were used as queries in a BLAST search against the durum wheat genome in the InterOmics (https://www.interomics.eu/) Svevo portal website. The output of this search was the hit match corresponding to the marker with the physical position. These positions were compared in the Genome Annotation Viewer (http://d-gbrowse.interomics.eu) to those obtained in the same way for QTLs associated with the traits considered in the present study.

## Results

### Phenotypic diversity

The field trials of tetraploid wheat accessions were carried out in two years at two contrasting environments in Kazakhstan, in the northern and south-eastern regions of the country. One hundred and eighty-four (184) accessions comprised of 122 durum wheat varieties (DWV), 55 domesticated tetraploid wheats (DTW) and 7 wild emmer accessions (WEA) (S1 Table) that were evaluated for the following agronomic traits: HD, SMT, NFS, NKS, PH, SL, TKW and YPP. Pearson correlation coefficient analysis of YPP evidenced weak positive correlations between two years within geographically different experimental sites (Table 1).

**Table 1:** Pearson’s coefficient of correlation in a world tetraploid wheat collection of 184 accessions evaluated for YPP in four environments.

	SEK18	SEK19	NK18
SEK19	0.25***		
NK18	0.12^ns^	0.13^ns^	
NK19	0.14*	0.35***	0.34***

SEK18, South-East Kazakhstan, 2018 growing year; SEK19, South-East Kazakhstan, 2019 growing year; NK18, North Kazakhstan, 2018 growing year; NK19, North Kazakhstan, 2019 growing year

ns, not significant

*significant at *P* ≤ 0.05

***significant at *P* ≤ 0.001

Since most of the durum wheat grows in the Northern territories of the country, the separate correlation analyses for averaged NK18 and NK19 field data were performed using Pearson’s coefficient. The yield evaluation in two environments of Northern Kazakhstan was not significantly different, although TKW in 2019 was significantly higher in comparison with 2018 (*P* < 0.0001). In the DWV and WDA (55 DTW + 7 WEA) sets, YPP was strongly and moderately correlated with NFS, respectively (Tables 2 and 3), while other traits showed no correlation or weak correlations (from 0.20 to 0.39).

**Table 2:** Pearson’s correlation indices between traits in the NK18 and NK19 field trials in the durum wheat sub-group.

	HD	SMT	PH	SL	NFS	NKS	TKW
SMT	-0.52****						
PH	0.21**	0.19*					
SL	0.27**	-0.04^ns^	0.34****				
NFS	-0.15^ns^	0.18*	0.14^ns^	0.13^ns^			
NKS	0.09^ns^	0.07^ns^	0.002^ns^	0.11^ns^	0.14^ns^		
TKW	-0.11^ns^	0.29***	0.07^ns^	0.05^ns^	0.11^ns^	-0.14^ns^	
YPP	0.23**	0.01^ns^	0.16^ns^	0.27**	0.69****	0.30***	0.36****

ns, not significant

*–significant at *P* ≤ 0.05

**–significant at *P* ≤ 0.01

***–significant at *P* ≤ 0.001

****–significant at *P* ≤ 0.0001; HD, heading date; SMT, seed maturation time; PH, plant height; SL, spike length; NFS, number of fertile spikes; NKS, number of kernels per spike; TKW, thousand kernel weight; YPP, yield per plant

**Table 3:** Pearson’s correlation indices between traits in NK18 and NK19 using wild and domesticated accessions.

	HD	SMT	PH	SL	NFS	NKS	TKW
SMT	-0.21^ns^						
PH	0.43***	0.11^ns^					
SL	0.31**	-0.17^ns^	0.37**				
NFS	-0.0009^ns^	0.02^ns^	-0.28*	0.04^ns^			
NKS	0.46****	-0.04^ns^	0.26*	-0.0003^ns^	-0.20^ns^		
TKW	0.03^ns^	0.30*	0.42***	0.13^ns^	-0.30*	-0.03^ns^	
YPP	0.28*	-0.06^ns^	0.07^ns^	0.006^ns^	0.51****	0.14^ns^	0.23^ns^

ns, not significant

*–significant at P ≤ 0.05

**–significant at *P* ≤ 0.01

***–significant at *P* ≤ 0.001

****–significant at *P* ≤ 0.0001; HD, heading date; SMT, seed maturation time; PH, plant height; SL, spike length; NFS, number of fertile spikes; NKS, number of kernels per spike; TKW, thousand kernel weight; YPP, yield per plant

The yield performance in both sets of accessions was compared to the YPP of “Damsinskaya yantarnaya” (DY), which is the standard durum wheat cultivar in Northern Kazakhstan. It was determined that the average of YPP in DWV (2.9 g ± 0.57) was higher and in WDA lower (2.4 g ± 0.6) in comparison to DY (2.6 g ± 0.67) (Table 4). In total, 80 accessions of the DWV and 18 accessions of WDA showed higher YPP values than the local standard. In other traits the comparison between DWV with DY showed that HD is shorter (up to 6 days), SMT is longer (up to 7 days) and PH is higher (19.3 cm ± 4) in DY, while for other traits no significant differences were detected. Similar differences for HD and SMT were recorded for comparative analyses of the WDA and DY. However, WDA was higher than DWV, and SL was 1.7 cm shorter in DWV (Table 4).

**Table 4:** Mean values of agronomic traits in the two collection sets evaluated in Northern Kazakhstan.

Traits	DWV	WDA	Standard cultivar (DY)
HD (days)	45.9 ± 2.2	47.1 ± 3.1	40.1 ± 2.2
SMT (days)	44.0 ± 5.8	44.8 ± 7.0	50.9 ± 3.9
PH (cm)	63.0 ± 9.6	85.3 ± 11.6	93.0 ± 4.0
SL (cm)	6.0 ± 0.7	7.7 ± 1.7	6.2 ± 0.4
NFS	1.8 ± 0.3	1.8 ± 0.5	2.3 ± 0.5
NKS	34.9 ± 4.5	29.1 ± 6.5	31.3 ± 2.1
TKW (g)	46.8 ± 4.1	46.7 ± 8.4	50.7 ± 6.7
YPP (g)	2.9 ± 0.6	2.4 ± 0.6	2.6 ± 0.7

DWV, durum wheat varieties; WDA, wild and domesticated accessions; HD, heading date; SMT, seed maturation time; PH, plant height; SL, spike length; NFS, number of fertile spikes; NKS, number of kernels per spike; TKW, thousand kernel weight; YPP, yield per plant; DY, ‘Damsynskaya Yantarnaya’ (local standard cultivar in Northern Kazakhstan)

GGE Biplot based on YPP also suggested that the collection of *T*. *durum* accessions has a higher value than DY in Northern Kazakhstan (S1D Fig). The second coordinate of the plot (42.0%) has also indicated that accessions of *T*. *dicoccum*, *T*. *carthlicum* and *T*. *turanicum* have potentials in yield improvement of durum wheat in the region. The GGE Biplot showed that heading date in the standard cultivar was distinctly different from remaining accessions (S1A Fig). In the case of SMT analysis, the GGE Biplot suggested that unlike accessions of *T*. *durum*, accessions of *T*. *dicoccum* and *T*. *dicoccoides* had nearly similar seed maturation time with DY (S1B Fig). Finally, GGE biplot evidenced a sharp contrast in PH between DY and the collection of *T*. *durum* (S1C Fig), as on average, DY was 26.2 cm taller than *T*. *durum* accessions. Among primitive and wild tetraploid species *T*. *turgidum*, *T*. *polonicum* and *T*. *dicoccoides* were in the range of PH in DY.

### Identification of marker-trait associations using TWC and DWV sets studied in two contrasting environments

**Fig 1:**
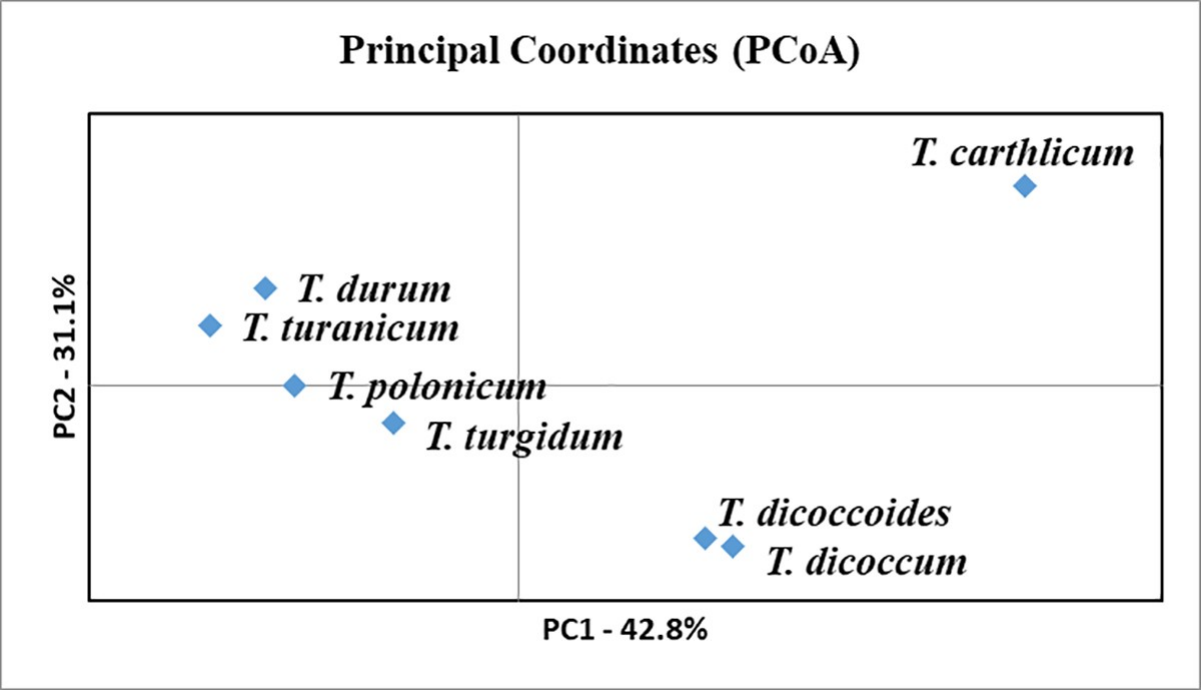
The principal coordinate analysis for seven wild and domesticated species in the world tetraploid wheat collection using 16,425 polymorphic SNPs and pairwise population values.

In this study, we used 16,425 polymorphic SNP markers obtained with 90K SNP assay [32]. The PCoA using the full set of polymorphic SNPs and pairwise population values suggested that *T*. *durum* and *T*. *turanicum* were genetically closer in comparison to other species (Fig 1). The first principal component in the PCoA (42.8%) clearly separated *T*. *durum* from free-threshing species *T*. *carthlicum* and hulled species *T*. *dicoccoides* and *T*. *dicoccum*. Further, the second principal component (31.1%) divided *T*. *durum* and *T*. *turanicum* from *T*. *polonicum* and *T*. *turgidum*.

**Fig 2:**
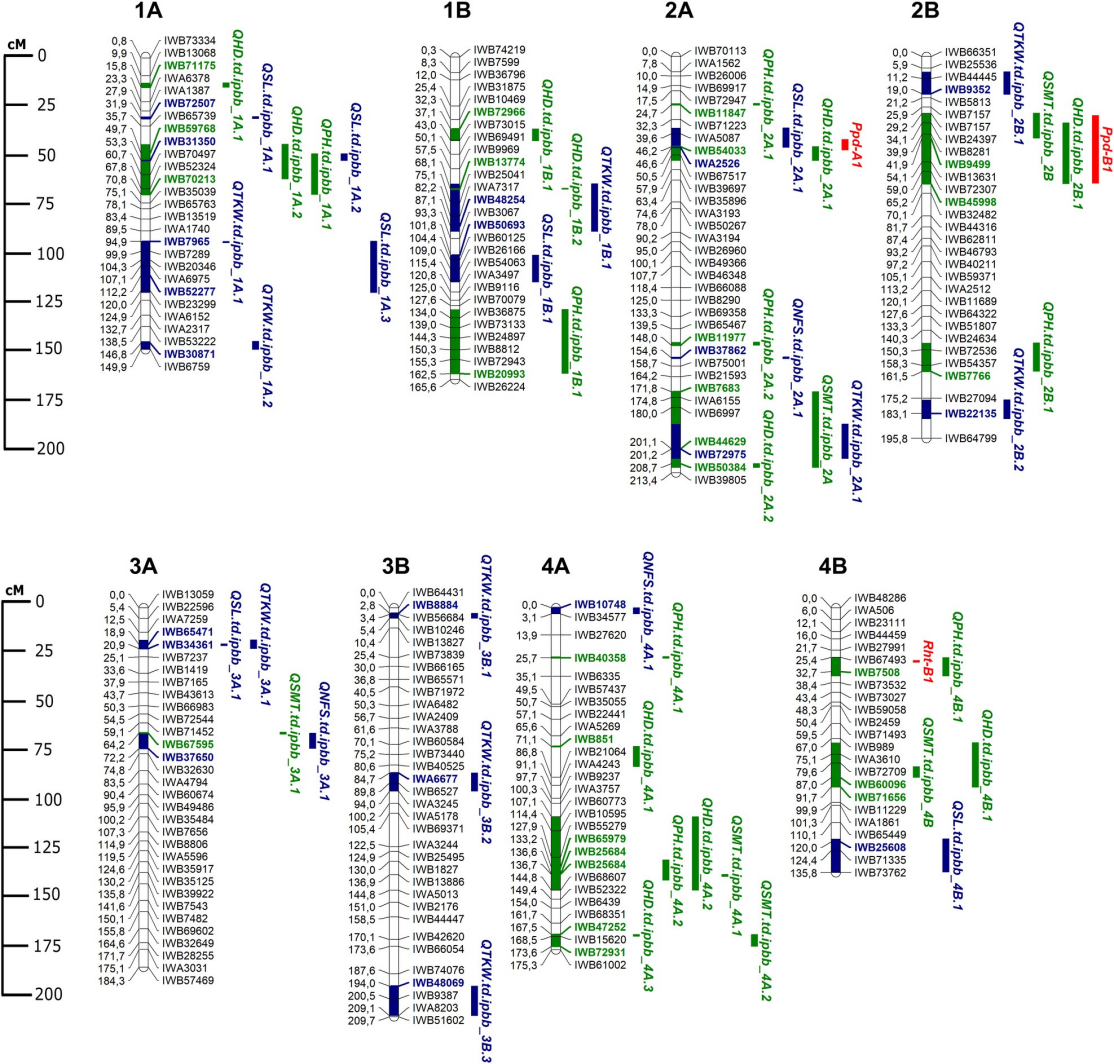
Schematic representation of identified Quantitative Trait Loci for agronomic traits in chromosomes 1A-4B of the wheat genomes using genome-wide association study. SNP marker positions for each chromosome were given based on [36]. QTLs for adaptive (green) and yield-related (blue) traits are shown on the right side of the chromosomes. QTL acronyms indicate the trait and chromosome. The most significant SNP markers are highlighted in color.

**Fig 3:**
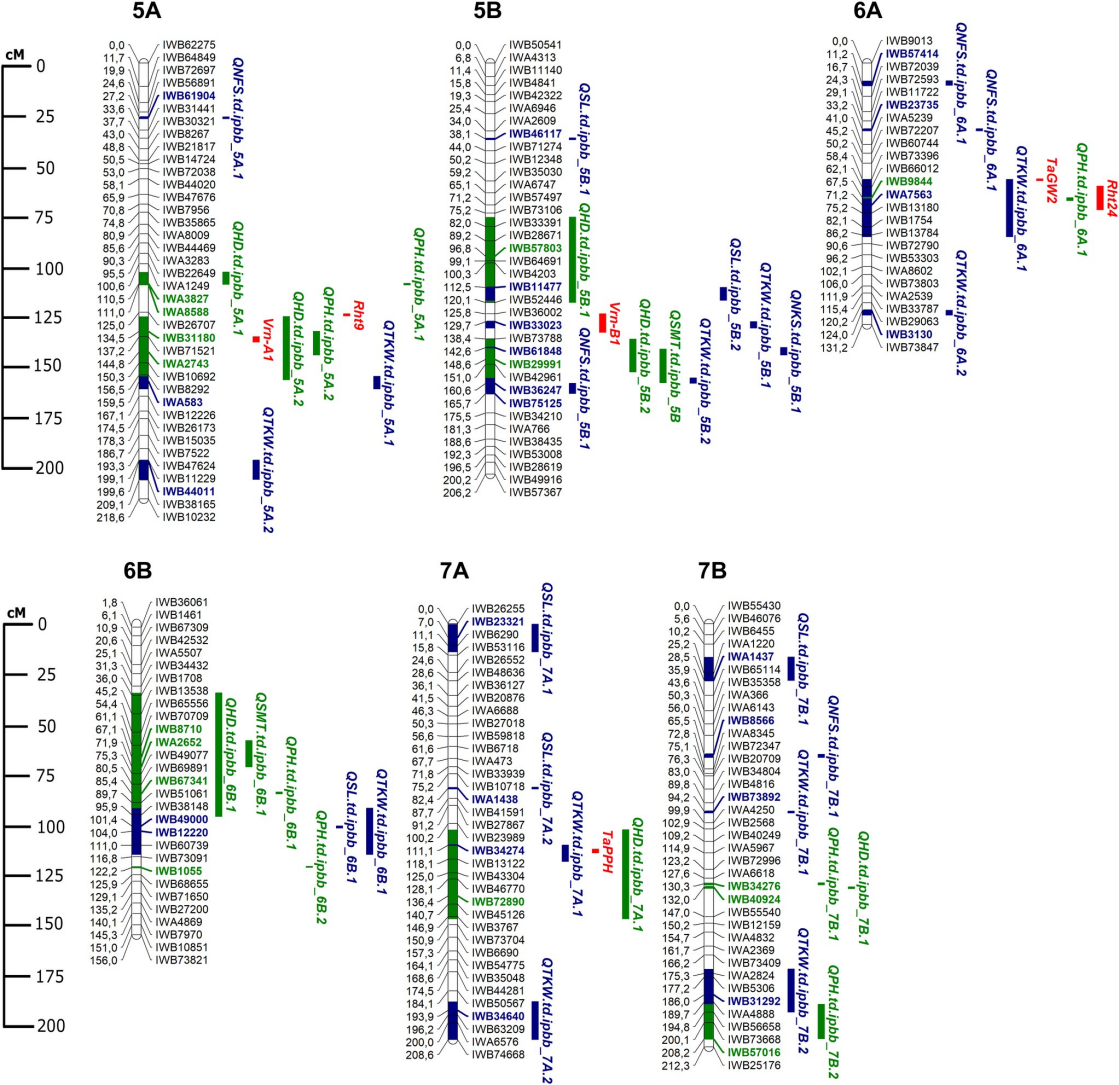
Schematic representation of identified Quantitative Trait Loci for agronomic traits in chromosomes 5A-7B of wheat genomes using genome-wide association study. SNP marker positions for each chromosome were assigned based on [36]. QTLs for adaptive (green) and yield-related (blue) traits are shown on the right side of the chromosomes. QTL names indicate the trait and chromosome. The most significant SNP markers are highlighted in color.

The clusterization results for the GWAS were adopted from the original study of the same collection, where the STRUCTURE allowed the revealing that the TWC set has the optimal K value 3, and the DWV set has K value 6 [14]. Overall, GWAS identified 108 significant marker-trait associations (MTAs) using the TWC and DWV sets. Moreover, 83 additional were detected in two or more environments using both (Tables 5 and 7). The GWAS of the TWC (n = 184) using seven agronomic traits has allowed the identification of 64 stable QTLs (Tables 5 and 6). The largest number of QTLs were identified for TKW (18), followed by HD (17) and SL (10) (Figs 2 and 3; Table 9). When the GWAS was performed only with the DWV set (n = 122), 59 stable QTLs were identified, hence the remaining five QTLs were detected in the WDA collection (Tables 7 and 8). In total, 40 identified QTLs were detected in both the TWC and DWV sets. The QTLs were localized in all chromosomes of the A and B genomes (Figs 2 and 3). Overall, the highest number of QTLs was identified on chromosome 1A, 4A and 5B (8 QTLs on each chromosome). Altogether, 45 QTLs were identified in the A genome and 38 QTLs in the B genome (Figs 2 and 3). Location-wise, 32 QTLs were identified in two contrasting regions (SEK and NK). Among the reported 83 QTLs, 39 were identified for adaptive traits and 43 for yield-related traits.

**Table 5:** The list of stable Quantitative Trait Loci (QTLs) controlling adaptive traits.

			TWC			DWV		
QTL	Chr	QTL (cM)^a^	*R*^*2*^ (%)	*P*-value	Env	*R*^*2*^ (%)	*P*-value	Env
*QHD*.*td*.*ipbb_1A*.*1*	1A	15.8	14.6	2.40E-06	3			
*QHD*.*td*.*ipbb_1A*.*2*	1A	44.9–62.8	9.8	7.79E-05	2			
*QHD*.*td*.*ipbb_1B*.*1*	1B	37.1–43.2	11.2	2.04E-05	2			
*QHD*.*td*.*ipbb_1B*.*2*	1B	67.6–68.1	13.8	1.083E-5	2			
*QHD*.*td*.*ipbb_2A*.*1*	2A	46.2–53.4	11.0	2.51E-05	1	13.3	1.13E-04	1
*QHD*.*td*.*ipbb_2A*.*2*	2A	208.7–210.8	15.5	2.93E-06	3			
*QHD*.*td*.*ipbb_2B*.*1*	2B	34–65.2	11.3	2.22E-05	2			
*QHD*.*td*.*ipbb_4A*.*1*	4A	71–71.4	10	6.45E-05	2	15.1	4.32E-05	1
*QHD*.*td*.*ipbb_4A*.*2*	4A	107.1–144.8	21.2	1.20E-08	4	15.4	4.40E-05	1
*QHD*.*td*.*ipbb_4A*.*3*	4A	167.5–168.5	13	4.91E-06	2			
*QHD*.*td*.*ipbb_4B*.*1*	4B	69.2–92	11	2.17E-05	2			
*QHD*.*td*.*ipbb_5A*.*1*	5A	104.9–111	9.3	1.29E-04	1	17.8	1.04E-05	2
*QHD*.*td*.*ipbb_5A*.*2*	5A	127.1–158.9	48.1	5.35E-06	1	21	1.90E-06	3
*QHD*.*td*.*ipbb_5B*.*1*	5B	77.3–120.1	15.5	3.22E-06	2			
*QHD*.*td*.*ipbb_6B*.*1*	6B	34.7–96.7	11.7	1.46E-05	3	12.8	1.74E-04	1
*QHD*.*td*.*ipbb_7A*.*1*	7A	103.3–148.1	9.9	6.83E-05	2			
*QHD*.*td*.*ipbb_7B*.*1*	7B	132–132.8	10.4	4.66E-05	1	13.4	1.27E-04	1
*QSMT*.*td*.*ipbb_2A*.*1*	2A	171.8–210.8	18.7	5.24E-07	2			
*QSMT*.*td*.*ipbb_2B*.*1*	2B	29–41.9	29.3	2.98E-10	2			
*QSMT*.*td*.*ipbb_3A*.*1*	3A	64.2	9.5	1.22E-04	2			
*QSMT*.*td*.*ipbb_4A*.*1*	4A	136.7–144.8	19.1	7.12E-08	2			
*QSMT*.*td*.*ipbb_4A*.*2*	4A	167.5–173.6	13.3	5.66E-05	3			
*QSMT*.*td*.*ipbb_4B*.*1*	4B	81.5–87	16.5	6.68E-07	2			
*QSMT*.*td*.*ipbb_5B*.*1*	5B	143.5–160.3	10.1	1.24E-04	2			
*QSMT*.*td*.*ipbb_6B*.*1*	6B	58.6–71.9	16.8	1.90E-06	2			
*QPH*.*td*.*ipbb_1A*.*1*	1A	49.8–70.8	7.1	9.60E-05	2	18	4.22E-05	1
*QPH*.*td*.*ipbb_1B*.*1*	1B	129.7–162.5	9.5	3.07E-05	1	21	1.49E-06	4
*QPH*.*td*.*ipbb_2A*.*1*	2A	24.7	14.7	1.08E-05	1	21	6.41E-06	3
*QPH*.*td*.*ipbb_2A*.*2*	2A	146.5–148				15	4.45E-05	2
*QPH*.*td*.*ipbb_2B*.*1*	2B	146.8–161.5				21	6.41E-06	3
*QPH*.*td*.*ipbb_4A*.*1*	4A	25.7	7.9	4.08E-05	1	21	6.41E-06	3
*QPH*.*td*.*ipbb_4A*.*2*	4A	129.3–139.7				21	6.41E-06	3
*QPH*.*td*.*ipbb_4B*.*1*	4B	28.5–35	7.6	6.51E-05	2	13.3	9.75E-05	2
*QPH*.*td*.*ipbb_5A*.*1*	5A	110.5–111.3				24.2	2.90E-06	4
*QPH*.*td*.*ipbb_5A*.*2*	5A	134.5–146.5				18	2.42E-05	2
*QPH*.*td*.*ipbb_6A*.*1*	6A	67.5–69.1				17.8	8.61E-06	3
*QPH*.*td*.*ipbb_6B*.*1*	6B	85.4				18.7	4.95E-05	3
*QPH*.*td*.*ipbb_6B*.*2*	6B	121.7–122.2				17.8	8.61E-06	3
*QPH*.*td*.*ipbb_7B*.*1*	7B	129.9–131				18.5	4.25E-05	2
*QPH*.*td*.*ipbb_7B*.*2*	7B	190.9–208.2				15	3.34E-05	2

Chr, Chromosome

^a^ QTL interval identified in this study; TWC, QTL identified in the World tetraploid wheat collection; DWV, QTL identified in Durum Wheat Varieties; Env, Number of environments

**Table 6:** The list of marker-trait associations related to plant adaptivity-related traits and information on their correspondence with previously reported QTL and known gene positions.

Trait	Marker	Chr	QTL (cM)^a^	QTL/Gene^b^	QTL; genetic position (cM)	QTL; physical position
HD	IWB71175	1A	15.8			
HD	IWB59768	1A	44.9–62.8	QTL [38]	18.4–60.7	18114055..461341317
HD	IWB72966	1B	37.1–43.2	QTL [38];	30.5–48.7;	88740151..398680744
				QTL [39]	36–52	157378023..445869269
HD	IWB13774	1B	67.6–68.1			
HD	IWB54033	2A	46.2–53.4	QTL/Gene [16]	42.6–59.2	
HD	IWB50384	2A	208.7–210.8	QTL [17];	196.1–206.1;	752518586..774769579
				QTL [40];	203.4–213.4;	762376432..774769579
				QTL [41]	204.3–209.3	764133958..774769579
HD	IWB45998	2B	34–65.2	QTL/Gene [39]	59.4–67.4	67807903..109351375
HD	IWB851	4A	71–71.4	QTL [17]	77.4–82.4	576057403..589990620
HD	IWB25684	4A	107.1–144.8	QTL [39]	134–142	636740842..692297825
HD	IWB47252	4A	167.5–168.5	QTL [17];	172.3–177.3;	722335492..736870383
				QTL [39]	170–178	717969013..736870383
HD	IWB71656	4B	69.2–92	QTL [39]	85–101	616758266..653894380
HD	IWA8588	5A	104.9–111	QTL [42]	103.4–136.3	465952726..535426236
HD	IWA2743	5A	127.1–158.9	Gene [43]		
HD	IWB57803	5B	77.3–120.1	Gene [39]		
HD	IWB8710	6B	34.7–96.7	QTL [38]	61.1–74.9	132052405..214321238
HD	IWB72890	7A	103.3–148.1	QTL [17]	133.8–143.8	577458282..637203668
HD	IWB40924	7B	132–132.8			
SMT	IWB44629	2A	171.8–210.8	QTL [40];	203.4–213.4	762376432..774769579
				QTL [40]	196.1–206.1	752518586..774769579
SMT	IWB9499	2B	29–41.9	QTL [39]	37.1–53.1	39637021..75854110
SMT	IWB67595	3A	64.2	QTL [17]	58.8–68.8	109507970..479941383
SMT	IWB25684	4A	136.7–144.8	QTL [39]	126–142	636740842..692297825
SMT	IWB72931	4A	167.5–173.6	QTL [17]	167.3–177.3	722335492..736870383
SMT	IWB60096	4B	81.5–87	QTL [38]	83.1–91.3	613218417..633215802
SMT	IWB29991	5B	143.5–160.3			
SMT	IWA2652	6B	58.6–71.9	QTL [40]	69.4–79.4	161768417..449108117
PH	IWB70213	1A	49.8–70.8			
PH	IWB20993	1B	129.7–162.5			
PH	IWB11847	2A	24.7			
PH	IWB11977	2A	146.5–148			
PH	IWB7766	2B	146.8–161.5	QTL [17]	155.5–165.5	729804343..749579012
PH	IWB40358	4A	25.7	QTL [17]	31.1–41.1	25942682..56387718
PH	IWB65979	4A	129.3–139.7	QTL [39]	126–142	636740842..692297825
PH	IWB7508	4B	28.5–35	QTL/Gene [44]	28–38.1	26783574..30374726
PH	IWA3827	5A	110.5–111.3	QTL [41]	NA	458989009..487823655
PH	IWB31180	5A	134.5–146.5	Gene [45]		
PH	IWB9844	6A	67.5–69.1	Gene [46]		
PH	IWB67341	6B	85.4			
PH	IWB1055	6B	121.7–122.2			
PH	IWB34276	7B	129.9–131	QTL [41]	130.1–132.6	613838400..630533637
				QTL [38]	132.7–140.7	599415770..648781988
PH	IWB57016	7B	190.9–208.2			

Chr, Chromosome

^a^ QTL interval identified in this study

^b^ The previously reported QTLs or genes within the same chromosomal regions with reference

**Table 7:** The list of identified Quantitative Trait Loci (QTLs) controlling yield-related traits.

			TWC			DWV		
QTL	Chr	QTL (cM)^a^	*R*^*2*^ (%)	*P*-value	Env	*R*^*2*^ (%)	*P*-value	Env
*QSL*.*td*.*ipbb_1A*.*1*	1A	31.9	9.8	1.33E-04	2			
*QSL*.*td*.*ipbb_1A*.*2*	1A	49.8–53.3	14.8	4.46E-06	2	44.9	4.65E-11	2
*QSL*.*td*.*ipbb_1A*.*3*	1A	94.7–121.1	10.4	7.87E-05	2	19.5	2.78E-05	3
*QSL*.*td*.*ipbb_1B*.*1*	1B	101.8–115.7				18.5	8.43E-06	2
*QSL*.*td*.*ipbb_2A*.*1*	2A	36.6–46.6	14.6	2.80E-05	1	17.3	1.56E-05	1
*QSL*.*td*.*ipbb_3A*.*1*	3A	18.9	11.6	5.68E-05	1	32.7	1.05E-07	2
*QSL*.*td*.*ipbb_4B*.*1*	4B	118.5–135.5	10.7	6.19E-05	1	12.2	1.83E-04	1
*QSL*.*td*.*ipbb_5B*.*1*	5B	38.1				15.9	3.16E-05	2
*QSL*.*td*.*ipbb_5B*.*2*	5B	112.5–119	13.2	7.63E-05	2			
*QSL*.*td*.*ipbb_6B*.*1*	6B	101.4–102.5	9.6	3.50E-05	2	12.8	1.57E-04	1
*QSL*.*td*.*ipbb_7A*.*1*	7A	0.3–14.2	9.7	1.69E-04	1	15.9	3.32E-05	1
*QSL*.*td*.*ipbb_7A*.*2*	7A	82.4				28.3	6.55E-08	2
*QSL*.*td*.*ipbb_7B*.*1*	7B	16.7–28.5	13.2	1.30E-05	1	27.6	1.11E-07	2
*QNFS*.*td*.*ipbb_2A*.*1*	2A	154.6	14.7	3.93E-06	1	24	4.18E-06	2
*QNFS*.*td*.*ipbb_3A*.*1*	3A	64.3–72.2	12.4	7.81E-06	1	16.2	1.21E-04	2
*QNFS*.*td*.*ipbb_4A*.*1*	4A	0–3.1	14	4.63E-06	1	23.5	4.31E-06	2
*QNFS*.*td*.*ipbb_5A*.*1*	5A	27.2				23.5	6.82E-07	2
*QNFS*.*td*.*ipbb_5B*.*1*	5B	160.6–165.7	9.5	1.76E-04	1	12.6	1.69E-04	1
*QNFS*.*td*.*ipbb_6A*.*1*	6A	8.9–11.2				30	3.06E-08	2
*QNFS*.*td*.*ipbb_6A*.*2*	6A	33.2	12.6	1.16E-05	1	19.7	2.90E-05	2
*QNFS*.*td*.*ipbb_7B*.*1*	7B	65.5–67.1				17.3	1.56E-05	2
*QNKS*.*td*.*ipbb_5B*.*1*	5B	142.6–146.5	11.1	5.6E-05	2			
*QTKW*.*td*.*ipbb_1A*.*1*	1A	94.9–95.5				15.4	1.60E-04	2
*QTKW*.*td*.*ipbb_1A*.*2*	1A	146.1–150.2	18.6	4.29E-07	1	15.4	1.50E-04	1
*QTKW*.*td*.*ipbb_1B*.*1*	1B	65.2–89.7	20.9	7.70E-08	3	19.4	3.38E-05	1
*QTKW*.*td*.*ipbb_2A*.*1*	2A	188.5–206.2	18.3	4.87E-07	2	20.4	2.52E-05	2
*QTKW*.*td*.*ipbb_2B*.*1*	2B	7.9–19.4	12.3	6.98E-06	1	16.6	1.26E-04	1
*QTKW*.*td*.*ipbb_2B*.*2*	2B	176–185.8	18.9	3.10E-07	1	37.6	1.17E-08	2
*QTKW*.*td*.*ipbb_3A*.*1*	3A	16.5–20.9	20.3	1.72E-07	1	13.5	1.96E-04	1
*QTKW*.*td*.*ipbb_3B*.*1*	3B	2.8–5.4	18.7	6.65E-07	2			
*QTKW*.*td*.*ipbb_3B*.*2*	3B	84.7–94	12.9	2.71E-05	1	15.6	1.77E-04	1
*QTKW*.*td*.*ipbb_3B*.*3*	3B	194–209.1				22.5	1.63E-06	1
*QTKW*.*td*.*ipbb_5A*.*1*	5A	157.1–163.5	14.5	7.77E-06	2	20.7	1.86E-05	3
*QTKW*.*td*.*ipbb_5A*.*2*	5A	199.1–208.8	14.5	7.77E-06	1	17.5	8.81E-05	2
*QTKW*.*td*.*ipbb_5B*.*1*	5B	129.7–132.9	18.9	4.14E-07	1	12.6	1.38E-04	1
*QTKW*.*td*.*ipbb_5B*.*2*	5B	157.9–160.6	11.3	3.45E-05	2			
*QTKW*.*td*.*ipbb_6A*.*1*	6A	58.4–87.1	20.3	1.25E-07	1	15.6	1.38E-04	1
*QTKW*.*td*.*ipbb_6A*.*2*	6A	124–126.5	9.7	1.42E-04	1	22.3	8.50E-06	2
*QTKW*.*td*.*ipbb_6B*.*1*	6B	92.6–115.8	18.8	3.15E-07	2	19.8	2.91E-05	1
*QTKW*.*td*.*ipbb_7A*.*1*	7A	111.1–119.3				19	7.45E-06	3
*QTKW*.*td*.*ipbb_7A*.*2*	7A	189.5–208.6	18.6	3.64E-07	3			
*QTKW*.*td*.*ipbb_7B*.*1*	7B	94.2	13.6	1.54E-05	1	24.4	1.94E-06	2
*QTKW*.*td*.*ipbb_7B*.*2*	7B	173.2–194.8	18.7	3.27E-07	1	15.9	1.16E-04	1

Chr, Chromosome

^a^ QTL interval identified in this study; TWC, QTL identified in the World tetraploid wheat collection; DWV, QTL identified in Durum Wheat Varieties; Env, Number of environments

**Table 8:** The list of marker-trait associations related to yield-related traits and information on their correspondence with previously reported QTL and known gene positions.

Trait	Marker	Chr	QTL (cM)^a^	QTL/Gene^b^	QTL; genetic position (cM)	QTL; physical position
SL	IWB72507	1A	31.9			
SL	IWB31350	1A	49.8–53.3			
SL	IWB52277	1A	94.7–121.1			
SL	IWB50693	1B	101.8–115.7			
SL	IWA2526	2A	36.6–46.6			
SL	IWB65471	3A	18.9			
SL	IWB25608	4B	118.5–135.5			
SL	IWB46117	5B	38.1			
SL	IWB11477	5B	112.5–119			
SL	IWB49000	6B	101.4–102.5			
SL	IWB23321	7A	0.3–14.2			
SL	IWA1438	7A	82.4			
SL	IWA1437	7B	16.7–28.5			
NFS	IWB37862	2A	154.6	QTL [47];	149.7–157.5;	697755950..712300489
				QTL [47]	151.7–159.6	698299250..712300489
NFS	IWB37650	3A	64.3–72.2	QTL [47];	69–76.8;	479956967..513797889
				QTL [47];	69.4–77.2;	479956967..513797889
				QTL [47]	69.6–77.5	479956967..513797889
NFS	IWB10748	4A	0–3.1			
NFS	IWB61904	5A	27.2			
NFS	IWB75125	5B	160.6–165.7	QTL [23]	141.6-NA	601246358..671280099
NFS	IWB57414	6A	8.9–11.2			
NFS	IWB23735	6A	33.2			
NFS	IWB8566	7B	65.5–67.1			
NKS	IWB61848	5B	142.6–146.5	QTL [47]	139.8–145.5	540387574..618978053
TKW	IWB7965	1A	94.9–95.5	QTL [39]	88.4–104.4	508236051..535156860
TKW	IWB9191	1A	146.1–150.2			
TKW	IWB48254	1B	65.2–89.7	QTL [42]	37.4–113.5;	33986133..594220377
				QTL [12]	53.9–57.6	473830872..473830942
TKW	IWB72975	2A	188.5–206.2	QTL [42];	201.1–210.8;	761215833..775446234
				QTL [48]	200.7–212.1;	753056610..775446234
				QTL [12]	181.2	737689634..737689537
TKW	IWB9352	2B	7.9–19.4			
TKW	IWB22135	2B	176–185.8	QTL [42];	191.9–193.6;	765305837..789411430)
				QTL [49]	181.6–187.6	772773642..782865134
TKW	IWB34361	3A	16.5–20.9			
TKW	IWB8884	3B	2.8–5.4	QTL [23];	1.1–4.9;	5604..5105605
				QTL [20]	4.2–12.5	5604..15077012
TKW	IWA6677	3B	84.7–94			
TKW	IWB48069	3B	194–209.1	QTL [23];	205.1;	796469438..827076832
				QTL [12];	178.6–194.6;	792002102..820343401
				QTL [39];	178.6–194.6;	792002102..820343401
				QTL [39];	178.6–194.6;	792002102..820343401
				QTL [12]	186.6	803216997..803216897
				QTL [39]	175.6–191.6	788010763..816570216
TKW	IWA583	5A	157.1–163.5			
TKW	IWB44011	5A	199.1–208.8			
TKW	IWB33023	5B	129.7–132.9			
TKW	IWB36247	5B	157.9–160.6			
TKW	IWA7563	6A	58.4–87.1	Gene [50]		
				QTL [12]	52.6	305092950..305093050
TKW	IWB3130	6A	124–126.5	QTL [23]	121.2-NA	598732579..608245286
TKW	IWB12220	6B	92.6–115.8	QTL [51];	104–112.9;	619901793..636619393
				QTL [52];	100.9–111;	621762855..636619393
				QTL [12]	97.5–113.5	601135266..645996827
				QTL [12]	105.5	628763171..628763073
TKW	IWB34274	7A	111.1–119.3	Gene [50]		
TKW	IWB34640	7A	189.5–208.6	QTL [12]	181.5–197.5	694638997..717853890
TKW	IWB73892	7B	94.2	QTL [12];	96.7–112.7;	496126667..578606740
				QTL [52]	85.9–92.9	459321833..517442227
TKW	IWB10520	7B	173.2–194.8			

Chr, Chromosome

^a^ QTL interval identified in this study

^b^ The previously reported QTLs or genes within the same chromosomal regions with reference

**Table 9:** The number of identified Quantitative Trait Loci in TWC and DWV datasets.

	TWC				DWV				Overall	
	Total	NK	SEK	NK/SEK	Total	NK	SEK	NK/SEK	Total	New
All traits	64	15	26	23	59	3	45	11	83	38
HD	17		11	6	7	1	5	1	17	3
SMT	8		4	4					8	1
PH	5	1	2	2	15	1	6	8	15	7
SL	10	3	4	3	11	1	9	1	13	13
NFS	5	1	4		8		7	1	8	5
NKS	1			1					1	
TKW	18	10	1	7	18		18		21	9

TWC, Tetraploid wheat collection; DWV, Durum wheat varieties; Total, total number of QTLs; NK, North Kazakhstan; SEK, South-East Kazakhstan; NK/SEK, both regions; New, no confirmation in AM studies; HD, heading date; SMT, seed maturation time; PH, plant height; SL, spike length; NFS, number of fertile spikes; NKS, number of kernels per spike; TKW, thousand kernel weight

### Plant adaptation-related traits

Three key traits, HD, SMT, and PH, were evaluated separately as they directly connected with plant adaptation of studied accessions in two contrasting sites. The HD study allowed the identification of 17 QTLs revealed in TWC analysis and 7 in DWV sets that were also found in TWC (Table 9). The percent of explained phenotypic variation (*R*^2^) of each of those QTLs ranged from 10 to 48%, *P*-values of marker-trait associations ranged from a minimum of 1.74E-04 to a maximum significance of 1.20E-08. Interestingly, the QTL with peak SNP marker IWB54033 (2A, 46.2 cM) appeared to be associated with the photoperiod sensitivity *Ppd-A* gene on the chromosome 2A, and IWB45998 marker (2B, 65.2 cM) is located near the known gene *Ppd-B1* (Fig 2). The other expected matches were the locus *QHD*.*td*.*ipbb_5A*.*2* located in the vicinity of the *Vrn-A1* (S2B and S2E Fig) and the locus *QHD*.*td*.*ipbb_5B*.*1* located in the region of the *Vrn-B1* (Fig 3, Table 6).

Eight QTLs for SMT were detected in the TWC, and none in DWV sub-group (Figs 2 and 3, Table 9). Each of these QTL explained between 9 and 33% of the phenotypic variation. Except for QTL in 3AL, all other associations for SMT were co-localized with QTLs for HD. The strongest association with this trait showed IWB9499 (2B, 41.9 cM) with the significance of *P* < 2.98E-10 (Table 5).

The GWAS for PH allowed the identification of 15 QTLs spread on ten different chromosomes evenly located on the A and B genomes (Table 9). The phenotypic variation of each of these QTL ranged from 6 to 24%. Four of those QTLs showed relatively high *R*^*2*^ values (from 21 to 24%) and were observed on chromosomes 1B, 4A and 5A (Table 5). The analysis in studied environments suggested that IWB20993 on chromosome 1B (162.5 cM) (S2H and S2J Fig) and IWA3827 on chromosome 5A (110.5 cM) (S2K and S2L Fig) were most stable and recorded being significant in four environments (Table 5). The IWB20993 was the most significantly associated SNP marker with PH and identified both using TWC (*R*^*2*^, 9%) and DWV (*R*^*2*^, 21%) sets. The next most significant QTLs were identified on chromosome 4A, where IWB40358 (25.7 cM) and IWB65979 (133.2 cM) showed a strong MTA (*P* < 6.41E-06). The IWA3827 (5A, 110.5 cM) also showed strong MTA (*P* < 2.90E-06) in the DWV set. Notably, three QTLs, designated here as *QPH*.*td*.*ipbb_4B*.*1*, *QPH*.*td*.*ipbb_5A*.*2* and *QPH*.*td*.*ipbb_6A*.*1*, were matching their genetic positions with known genes, *Rht-B1*, *Rht9* and *Rht24*, respectively (Figs 2 and 3, Table 6).

### Yield-related traits

Five key agronomic traits directly related to yield (SL, NFS, NKS, TKW, and YPP) were considered for the analyses in this part of GWAS. However, no QTL was identified for YPP. The GWAS for SL has allowed the identification of 13 QTLs, and 8 of those QTLs were common both in TWC and DWV screenings (Table 9, Figs 2 and 3), suggesting that WDA significantly contributed to the identification of additional 5 QTLs. The most stable QTL (*QSL*.*td*.*ipbb_1A*.*3)* significant in three studied environments was identified on chromosome 1A (112.2 cM) that was detected in three environments (Table 7). The most significant QTL for this trait was observed on chromosome 7A (82.8 cM) in the DWV sub-set (*P* < 6.55E-08). Among eight QTLs identified for NFS in the DWV sub-set, five were detected in TWC (Table 9), suggesting that most significant QTLs located on chromosomes 6A (11.2 cM, *P* < 3.06E-08) and 5A (27.2 cM, P < 6.82E-07), respectively (Table 7).

The largest number of QTLs was identified for TKW, with 21 QTLs significant in two and more environments (Table 9). It was shown that 18 of those 21 QTLs were mapped in the DWV set, while the remaining three were identified in the TWC set (Table 7). Overall, 15 QTL regions were evidenced in both the TWC collection and DWV sub-set. In DWV analysis, the most stable QTLs were identified on chromosomes 5A (159.5 cM) and 7A (111.1 cM), both showing the significance in three environments (SEK18-1, SEK18-2, SEK18-m) (S3 Table). In TWC analysis, the QTLs on chromosomes 1B (87.1 cM) and 7A (193.9 cM) also recorded in three environments (SEK18-1; SEK18-m; NK19). The most significant association was recorded for IWB22135 (2B, 183.1 cM) that was identified in both sets of accessions, with an *R*^*2*^ of 37.6% in DWV subset study (Table 7).

## Discussion

A comparative assessment of the TWC set for yield in Northern and South-eastern regions of Kazakhstan, two sharply contrasting sites with different altitudes, soil quality and meteorological conditions (S2 Table), showed a rather unstable correlation in the two years (Table 1). Pearson’s correlation coefficient for yield performance in two years ranged from a non-significant correlation in 2018 to highly significant in 2019, with a weak correlation between SEK18 and NK19. Therefore, it is evident that complex traits, such as grain yield, are primarily governed by multiple genes and environmental factors. As most of the durum wheat in the country is grown in high-quality soil area of Northern Kazakhstan, the primary focus of this study was the Northern region. The NK18 and NK19 trials investigating eight major agronomic traits in the DWV sets study revealed that yield components were significantly correlated with HD and SMT, suggesting that plant adaptive traits play an important role in grain productivity in durum wheat. Despite sharp differences of the TWC and DWV sets with standard cultivar DY in averaged HD, SMT and PH values (Table 2), it was determined that 80 DWV and 18 WDA accessions showed higher average yield than in DY. One week earlier SMT, in comparison with DY, seems a particular value of the studied collection, as SMT positively correlated with TKW (Table 2). Also, average NKS in DWV over two years showed a higher value (to 3.6 g) than in DY. Therefore, a superior yield of DWV lines in Northern Kazakhstan indicates that the majority of durum wheat accessions evaluated in this study are beneficial for the improvement of local breeding programs targeting productivity.

GGE Biplot analyses for yield performance revealed that accessions of *T*. *dicoccum*, *T*. *carthlicum*, and *T*. *turanicum* also have potentials in yield improvement of durum wheat in the region (S1D Fig). In addition, among other wild tetraploid species, *T*. *turgidum*, *T*. *polonicum* and *T*. *dicoccoides* were in the range of PH in DY (S1C Fig). This finding is important, as local breeders consider a taller PH as one of the beneficial traits for better adaptability of breeding material in the region (Table 4).

GWAS of eight agronomic traits in TWC and DWV sets harvested in two contrasting regions. GWAS for identification of marker-trait associations (MTAs) in TWC and DWV sets harvested in four environments was conducted by analyzing seven agronomic traits. The entire collection was previously genotyped using 16,425 polymorphic SNPs [32]. Notably, the PCoA based on 16,425 SNPs suggested that among domesticated species, T. turanicum is most genetically close to T. durum (Fig 1). The GWAS has allowed the identification of 40 MTAs for three plant adaptation traits (HD, SMT and PH) and 43 MTAs for four yield-related traits (SL, NFS, NKS and TKW). The comparison of 83 identified MTAs with previously published reports in GWAS for durum wheat suggested that 38 MTAs are presumably novel (Table 9). The remaining 45 MTAs were previously identified based on field trials in other parts of the World. The large number of co-localized MTAs for the same analyzed traits confirms the validity of this study.

*The assessment of identified marker-traits associations for adaptive traits*. The analysis of yield performance in two different regions of the country suggested a strong influence of environmental conditions (Table 4), and the sensitivity of the accessions can be accounted by the effects of environmental factors at crucial growth phases, which determines the potential grain productivity [10]. Therefore, field tests of the well-studied collection in new environments may potentially lead to the identification of new significant MTAs. These discovered MTAs were particularly important for the Northern region tested site where over 85% of the durum wheat area in the country is located. In this analysis, 40 stable MTAs for adaptative traits comprised of 17 HD, 8 SMT and 15 PH associations. The observation of the list of 17 MTAs for HD (Table 5) suggests that five MTAs are presumable novel associations and were never reported previously (Table 9). The physical location of the most significant SNPs in those five MTAs indicated that four significant SNPs were located in the coding region and 3 SNPs in non-coding regions (S3 Table). In total, in the trials conducted in Northern territories, eight MTAs for HD were identified, including five presumably new highly significant MTAs (Figs 2 and 3, Table 9). Notably, the *QHD*.*td*.*ipbb_2B*.*1* was shown to correspond to *Ppd-B1* gene [53]. Also, *QHD*.*td*.*ipbb_5A*.*2* was located in the vicinity of the *Vrn-A1* and the *QHD*.*td*.*ipbb_5B*.*1* in the region of the *Vrn-B1* (Fig 3, Table 5).

Unlike in HD and SMT, the source of 15 identified MTAs for PH was the DWV set, and using the entire TWC set also helped in revealing five of those MTAs, suggesting that one-third of those associations was contributed by the WDA set (Table 6). Therefore, the WDA set is a rich source for discovering new genes that affect PH in tetraploid wheat. Out of nine presumably novel, MTAs for PH three MTAs were also found using TWC set, including one significant SNP in the MTA in the coding region, and SNPs in two MTAs in non-coding regions. The majority of 15 MTAs for PH, except one MTA in the Northern site and four MTAs in the South-east site, were revealed in both locations (S3 Table). Three out of five MTAs identified using the TWC set, *QPH*.*td*.*ipbb_4B*.*1* (*Rht-B1*), *QPH*.*td*.*ipbb_5A*.*2* (*Rht9*), *QPH*.*td*.*ipbb_6A*.*1* (*Rht24*), were observed in Northern station (Figs 2 and 3).

*The assessment of identified marker-trait associations for yield-contributing traits*. The evaluation of 43 MTAs for yield-contributing traits suggested that thirteen MTAs were identified for SL, eight for NFS, one for NKS, and 21 for TKW (Table 9). None of the MTA were mapped in positions of known specific genes that control these traits, except two TKW-associated genes (Table 7). In the first case, *QTKW*.*td*.*ipbb_6A*.*1* (58.5–87.1 cM) identified in this study was positioned in the vicinity of gene *TaGW2-6A* (a negative regulator of grain size and weight) [54, 36]. In the second instance, *QTKW*.*td*.*ipbb_7A*.*1* (111.1 cM) identified in this study was mapped in the position of *TaPPH-7A* (involved in chlorophyll degradation, further affects yield and quality of crops). [50] (Table 7).

A different result was recorded when MTAs in this study were compared with known QTL for these five traits in previously published reports. The SL is one of the fundamental traits in spike architecture, and not only directly influences grain yield [55], but also significantly associated with HD [56]. This study confirms the significant relationship between SL and HD in durum wheat (*P* < 0.001), and four out of thirteen MTAs for SL here were associated with HD in durum wheat (Figs 2 and 3). Notably, six out of thirteen MTAs for the SL in the TWC set and two MTAs in the DWV set were identified in the Northern Kazakhstan testing site and can be effectively targeted in local breeding programs. The comparative analysis for NFS, another important yield component, suggested that four of eight identified in this study MTAs were previously reported. One of the reported MTAs for NFS (*QNFS*.*td*.*ipbb_3A*.*1*) was also identified in the NK testing site with high significance (*P* < 0.0001). Likewise, the identified single MTA for NKS both in South-eastern and Northern territories was also previously reported [47].

The largest number of MTAs among all studied traits were identified in TKW (21 MTAs) (Table 9), which is known as an essential yield-contributing trait [57]. The comparative assessment of the physical location of identified MTA for TKW with previously reported QTL suggested that 11 out of 21 MTAs were presumably novel (Table 7). The majority of the identified MTAs (18) was revealed using the DWV set, and 17 using the TWC set. Interestingly, all MTAs using the DWV set were detected only in the South-east testing site, suggesting that the WDA set is a valuable source for a new MTA for TKW in the Northern testing site. Thus, the study was an additional contribution to the understanding of the genetic dissection of this complex trait. Obtained results would serve as a required prerequisite for forming and realization of specific breeding programs towards effective adaptation and increased productivity of durum wheat in Kazakhstan. Partially, this can be achieved by converting the identified SNP markers of 83 MTAs to reliable KASP (Kompetitive Allele Specific PCR) type of markers [58].

## Conclusions

Field trials of the world tetraploid wheat collection (n = 184), including 122 durum wheat accessions, in two different regions in Kazakhstan, showed an unstable yield correlation over two studied years (2018–2019). The study revealed that the majority of the collection showed high productivity in comparison with local durum wheat standard cultivar. In particular, it was shown that 80 DWV and 18 WDA accessions showed higher average yield than in DY, the standard cultivar for Northern Kazakhstan, where more than 80% of the durum wheat cultivated in the country. The field tests in the Northern site using eight major agronomic traits in the DWV sets revealed that yield components were significantly correlated with HD and SMT, suggesting that adaptative traits play an important role in grain productivity in durum wheat. Also, GGE Biplot analyses for yield performance revealed that accessions of *T*. *dicoccum*, *T*. *carthlicum* and *T*. *turanicum* also have potentials in yield improvement of durum wheat in the region.

The GWAS identified 40 MTAs for three adaptative traits (HD, SMT and PH) and 43 MTAs for four yield-related traits (SL, NFS, NKS and TKW). The comparison of 83 identified MTAs with previously published reports in GWAS for durum wheat suggested that 38 MTAs are presumably novel. The co-localization of a large number of MTAs with those previously published confirms the validity of the results of this study. The MTAs reported herewith will provide the opportunity to implement marker-assisted selection in ongoing durum wheat breeding projects targeting higher productivity in the region.

## Supporting information

S1 Fig GGE Biplot for key agronomic traits of tetraploid wheat collection harvested in Northern Kazakhstan. (DOCX)

S2 Fig Manhattan and Q-Q plots based on the analysis of field data from South-East and North Kazakhstan analyzed using the R GAPIT package. (DOCX)

S1 Table List of tetraploid wheat accessions. (XLSX)

S2 Table Meteorological and location data in two regions of Kazakhstan. (DOCX)

S3 Table The full list of identified stable quantitative trait loci using world tetraploid wheat collection. (XLSX)
