# Supplementary material for: Quantitative trait loci for agronomic traits in tetraploid wheat for enhancing grain yield in Kazakhstan environments
Source: PLoS One. 2020 Jun 23;15(6):e0234863. doi: 10.1371/journal.pone.0234863 (PMC7310741; doi:10.1371/journal.pone.0234863)
Supplement: S1 Fig — (DOCX) [file pone.0234863.s001.docx]

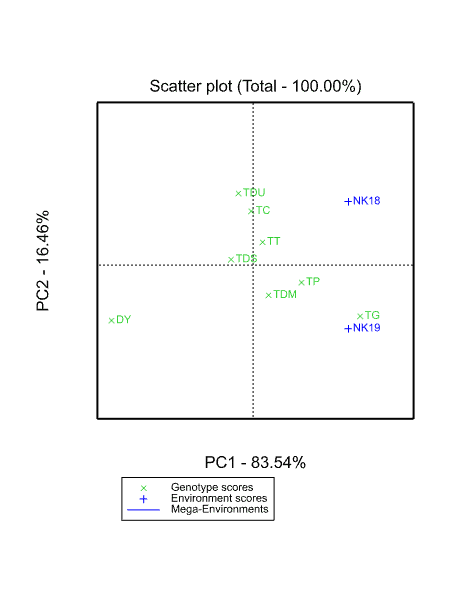

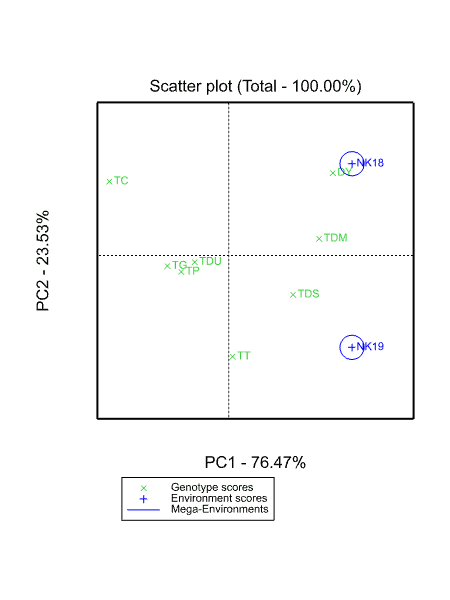


A B


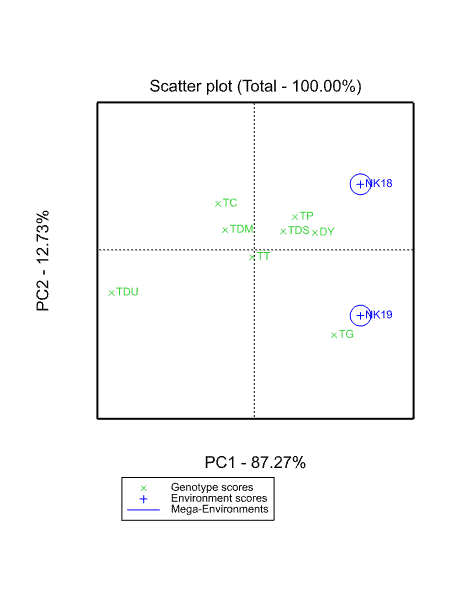

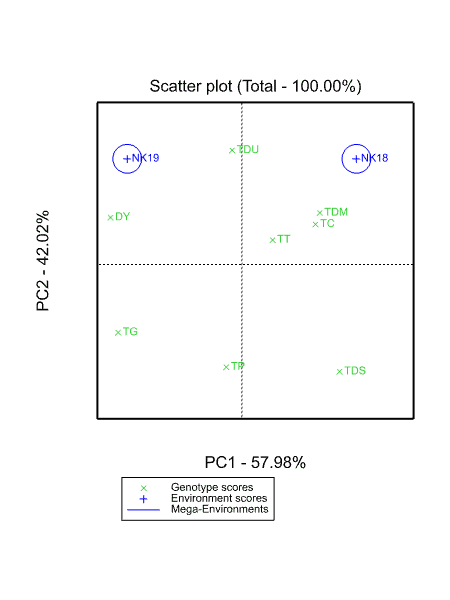


C D

**S1 Fig. GGE Biplot for key agronomic traits of tetraploid wheat collection harvested in Northern Kazakhstan**. A: Heading date (HD); B: Seed maturation time (SMT); C: Plant height (PH); D: Yield per plant (YPP); DY: Damsynskaya yantarnaya (check cultivar); TT: *T. turanicum*; TP: *T. polonicum*; TG: *T. turgidum*; TC: *T. carthlicum*; TDM: *T. dicoccum*; TDS: *T. dicoccoides*;

TDU: *T. durum*
