# Supplementary material for: Quantitative trait loci for agronomic traits in tetraploid wheat for enhancing grain yield in Kazakhstan environments
Source: PLoS One. 2020 Jun 23;15(6):e0234863. doi: 10.1371/journal.pone.0234863 (PMC7310741; doi:10.1371/journal.pone.0234863)
Supplement: S2 Fig — (DOCX) [file pone.0234863.s002.docx]

**Supplemental Figure S2. Manhattan and Q-Q plots based on the analysis of field data from South-East and North Kazakhstan analyzed using the R GAPIT package**

| 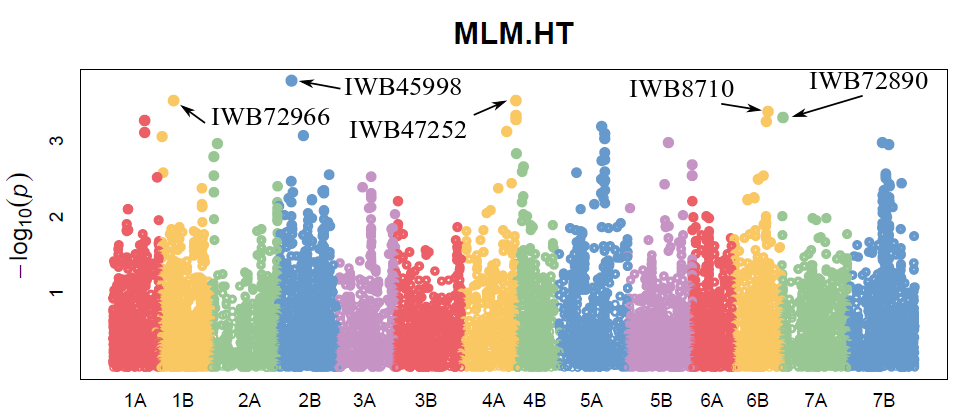 | 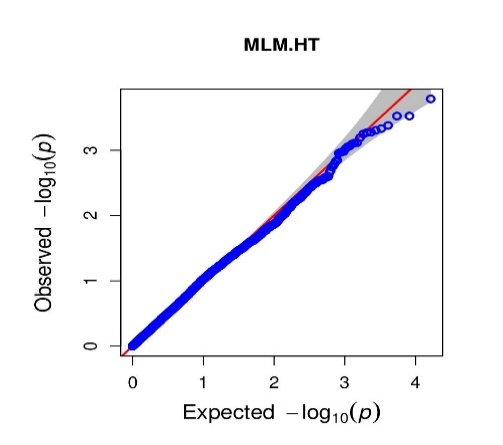 |
| --- | --- |
| A | B |

**S2 Fig 1 Heading date MTAs in SEK18 (TWC).** A) Manhattan plot. B) Q-Q plot. Vertical axes show the negative logarithm of the association *P*-value. Horizontal axes show chromosomes. Arrows indicate identified stable MTAs.

| 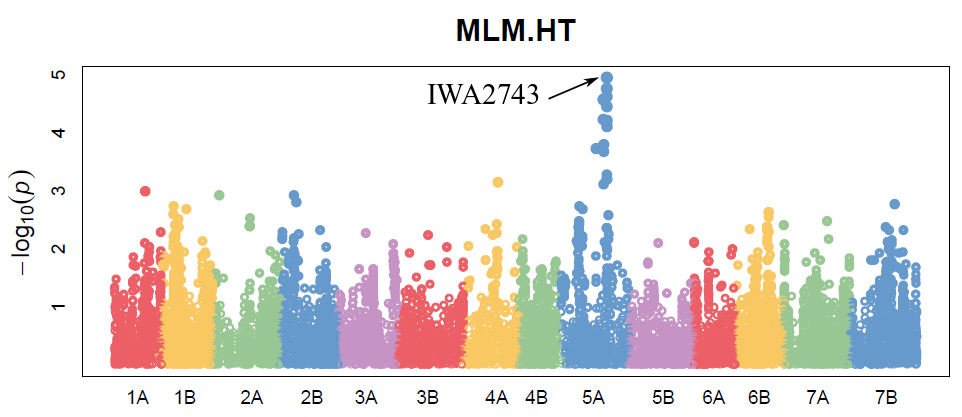 | 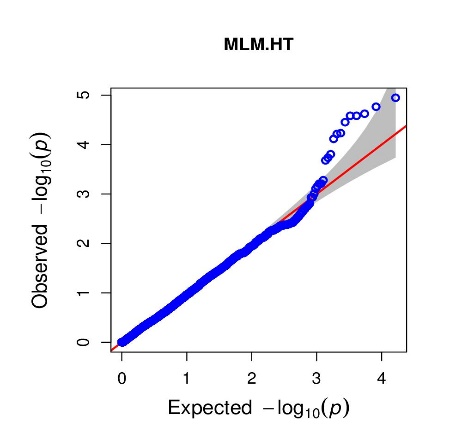 |
| --- | --- |
| A | B |

**S2 Fig 2 Heading date MTAs in SEK18 (DWV).** A) Manhattan plot. B) Q-Q plot. Vertical axes show the negative logarithm of the association *P*-value. Horizontal axes show chromosomes. Arrows indicate identified stable MTAs.

| 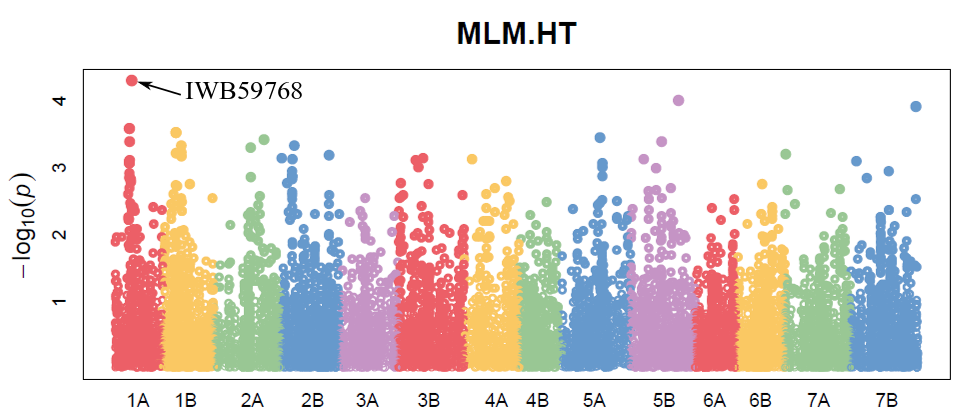 | 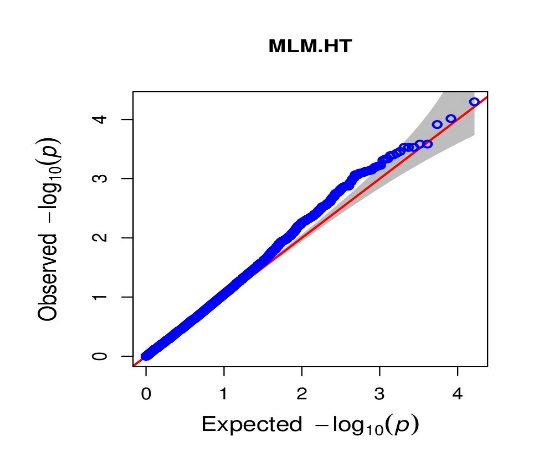 |
| --- | --- |
| A | B |

**S2 Fig 3 Heading date MTAs in SEK19 (TWC).** A) Manhattan plot. B) Q-Q plot. Vertical axes show the negative logarithm of the association *P*-value. Horizontal axes show chromosomes. Arrows indicate identified stable MTAs.

| 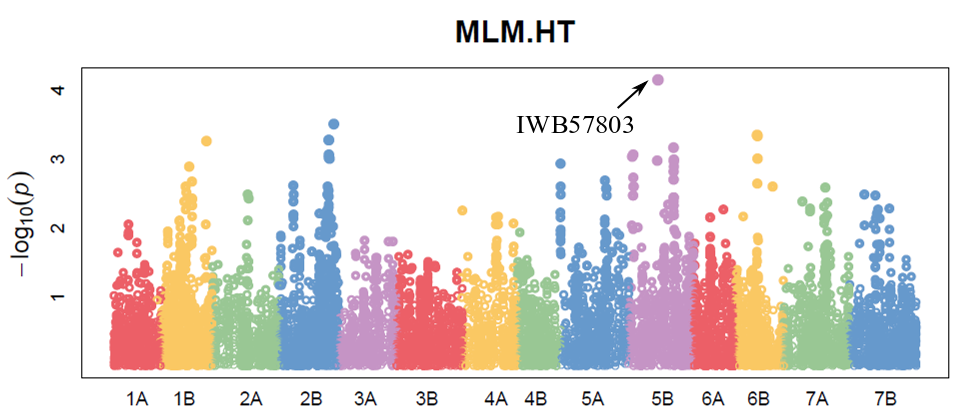 | 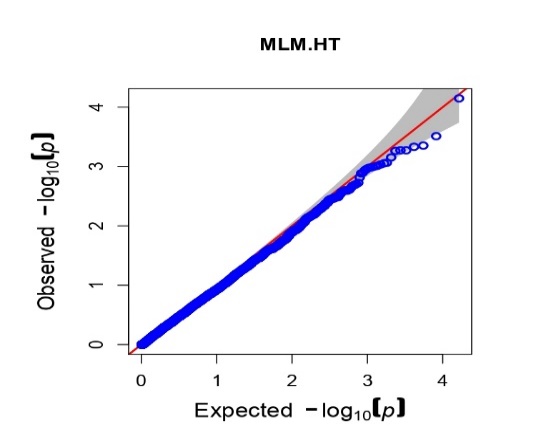 |
| --- | --- |
| A | B |

**S2Fig 4 Heading date MTAs in NK18 (DWV).** A) Manhattan plot. B) Q-Q plot. Vertical axes show the negative logarithm of the association *P*-value. Horizontal axes show chromosomes. Arrows indicate identified stable MTAs.

| 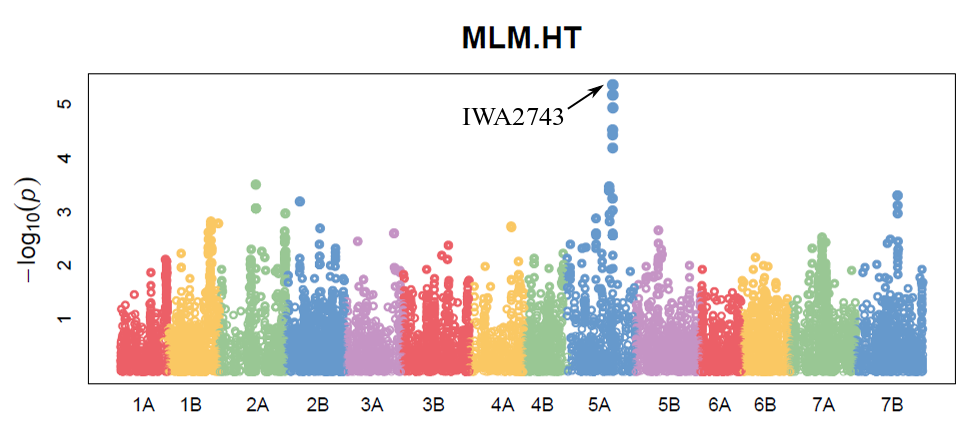 | 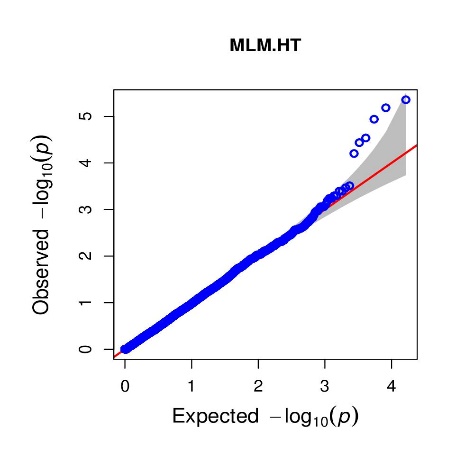 |
| --- | --- |
| A | B |

**S2 Fig 5 Heading date MTAs in NK19 (DWV).** A) Manhattan plot. B) Q-Q plot. Vertical axes show the negative logarithm of the association *P*-value. Horizontal axes show chromosomes. Arrows indicate identified stable MTAs.

| 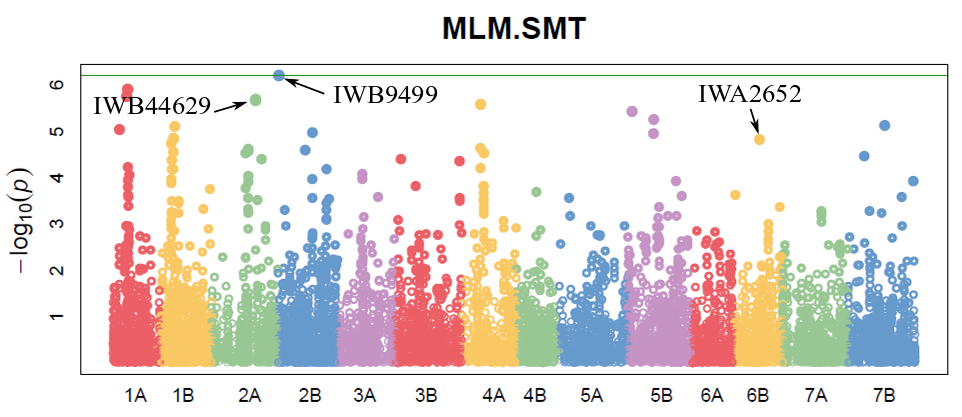 | 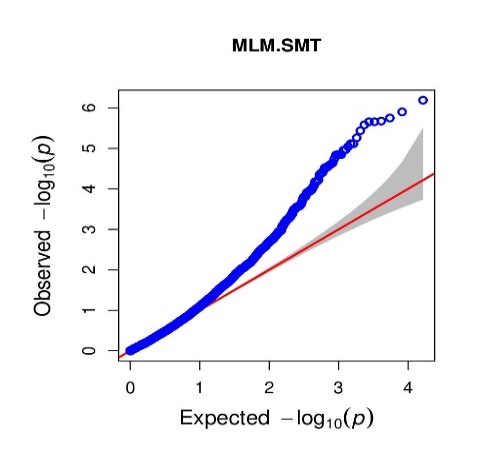 |
| --- | --- |
| A | B |

**S2 Fig 6 Seed maturation time MTAs in SEK19 (TWC).** A) Manhattan plot. B) Q-Q plot. Vertical axes show the negative logarithm of the association *P*-value. Horizontal axes show chromosomes. Arrows indicate identified stable MTAs.

| **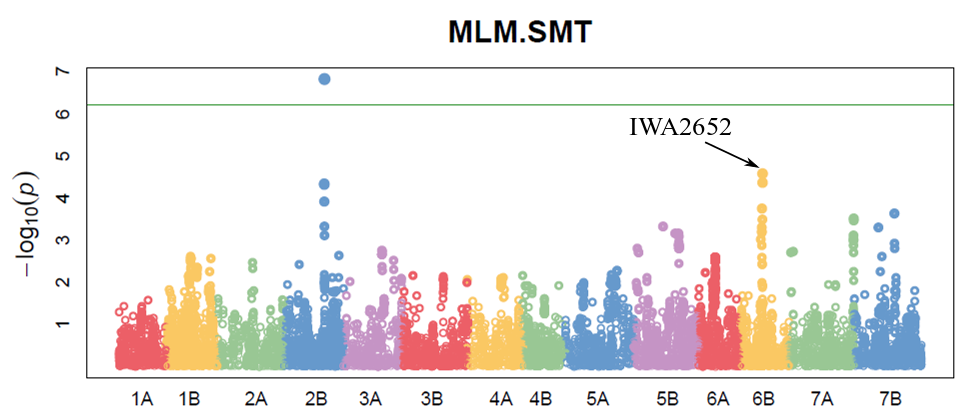** | **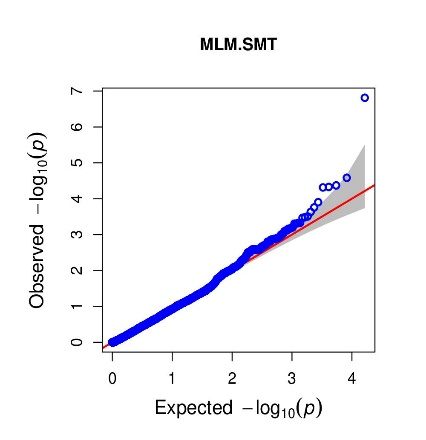** |
| --- | --- |
| A | B |

**S2 Fig 7 Seed maturation time MTAs in NK18 (DWV).** A) Manhattan plot. B) Q-Q plot. Vertical axes show the negative logarithm of the association *P*-value. Horizontal axes show chromosomes. Arrows indicate identified stable MTAs.

| 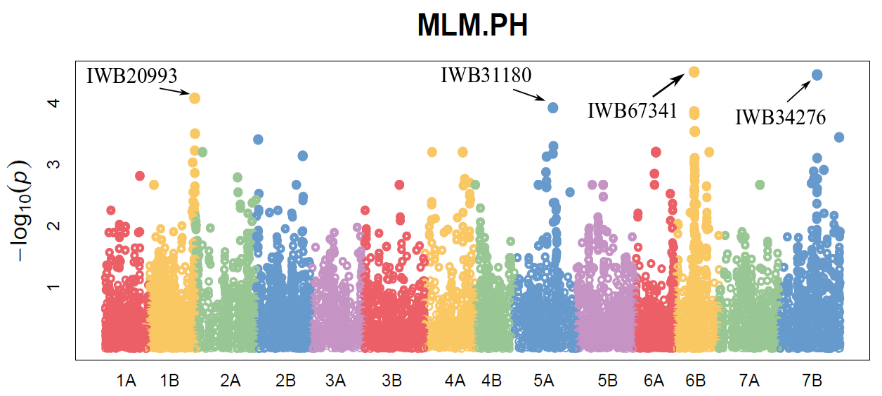 | 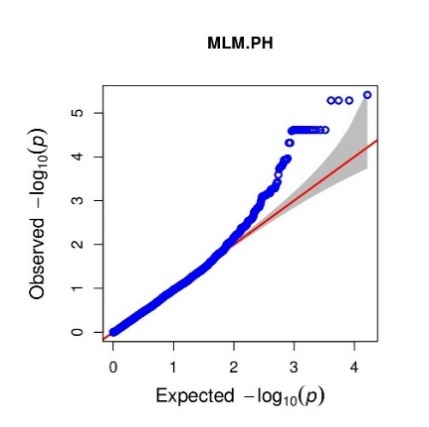 |
| --- | --- |
| A | B |

**S2 Fig 8 Plant height MTAs in NK18 (DWV).** A) Manhattan plot. B) Q-Q plot. Vertical axes show the negative logarithm of the association *P*-value. Horizontal axes show chromosomes. Arrows indicate identified stable MTAs.

| 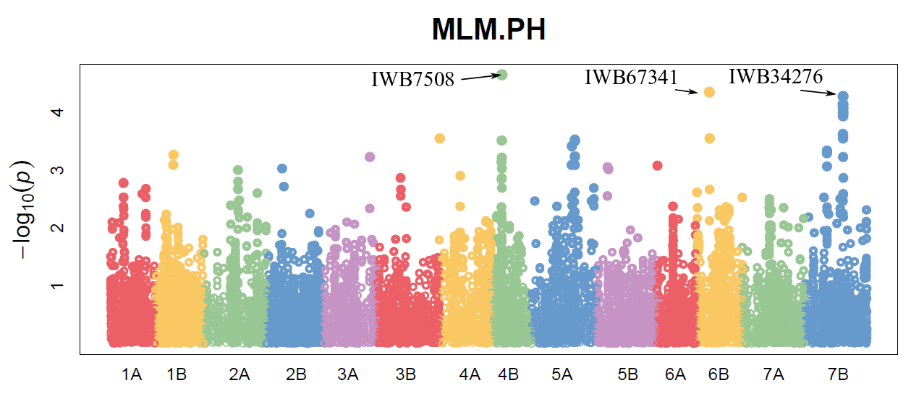 | 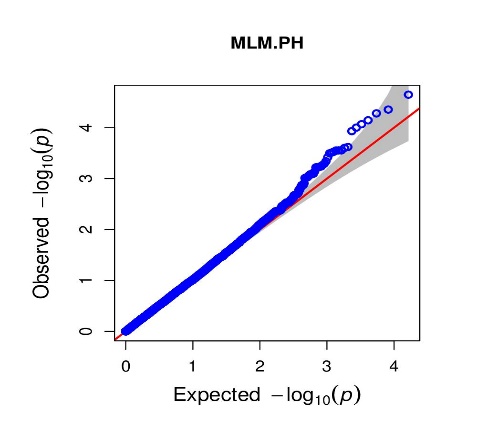 |
| --- | --- |
| A | B |

**S2 Fig 9 Plant height MTAs in NK19 (TWC).** A) Manhattan plot. B) Q-Q plot. Vertical axes show the negative logarithm of the association *P*-value. Horizontal axes show chromosomes. Arrows indicate identified stable MTAs.

| 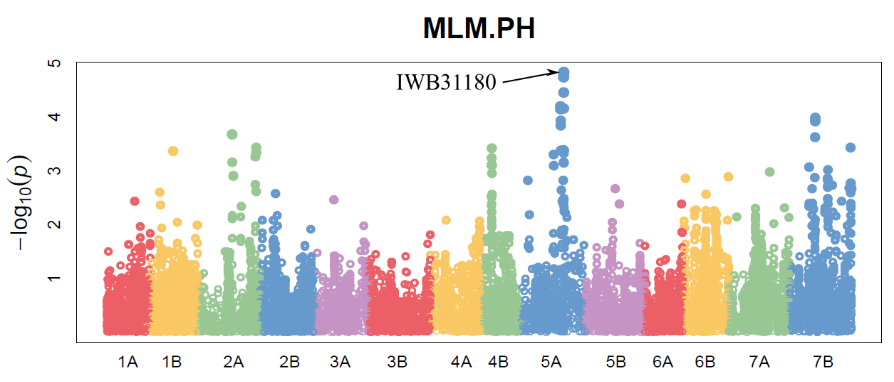 | 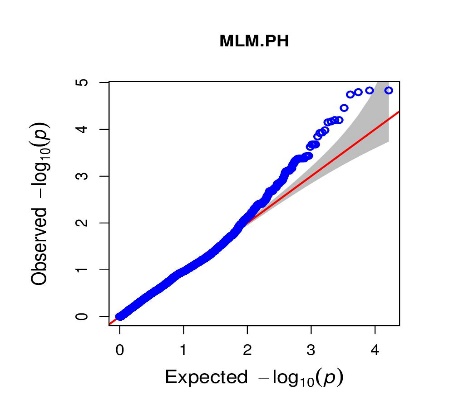 |
| --- | --- |
| A | B |

**S2 Fig 10 Plant height MTAs in NK19 (DWV).** A) Manhattan plot. B) Q-Q plot. Vertical axes show the negative logarithm of the association *P*-value. Horizontal axes show chromosomes. Arrows indicate identified stable MTAs.

| 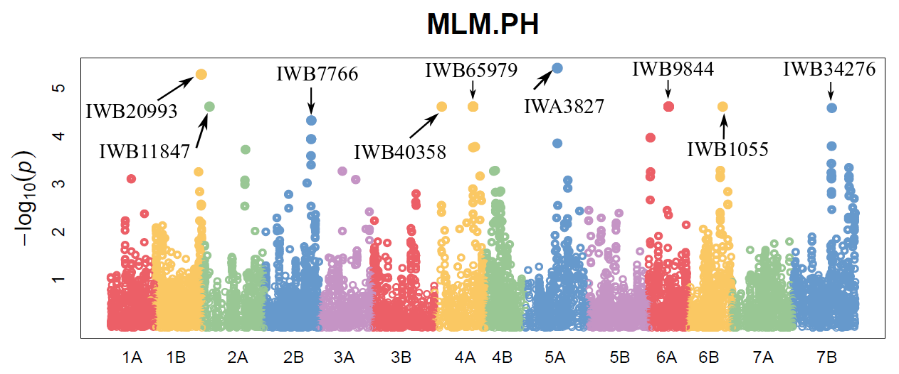 | 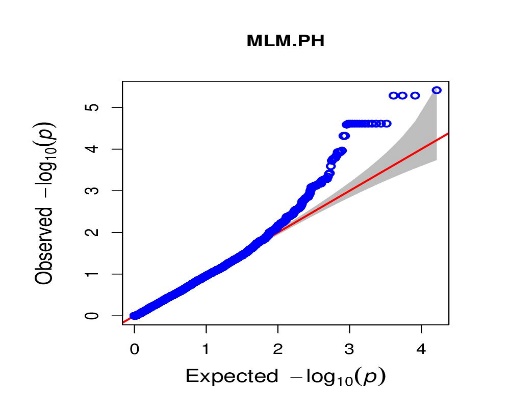 |
| --- | --- |
| A | B |

**S2 Fig 11 Plant height MTAs in SEK18 (DWV).** A) Manhattan plot. B) Q-Q plot. Vertical axes show the negative logarithm of the association *P*-value. Horizontal axes show chromosomes. Arrows indicate identified stable MTAs.

| 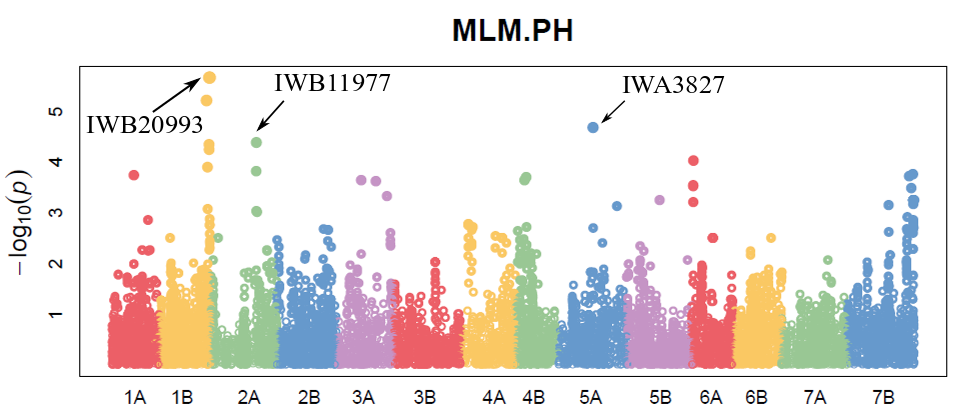 | 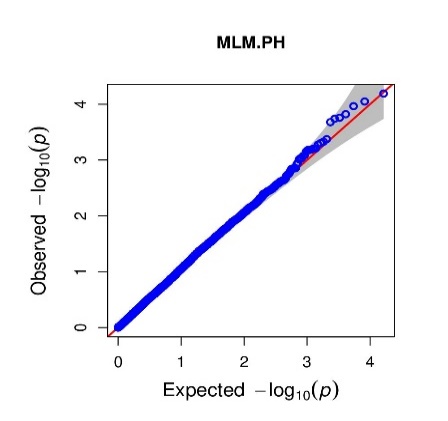 |
| --- | --- |
| A | B |

**S2 Fig 12 Plant height MTAs in SEK19 (DWV).** A) Manhattan plot. B) Q-Q plot. Vertical axes show the negative logarithm of the association *P*-value. Horizontal axes show chromosomes. Arrows indicate identified stable MTAs.

| **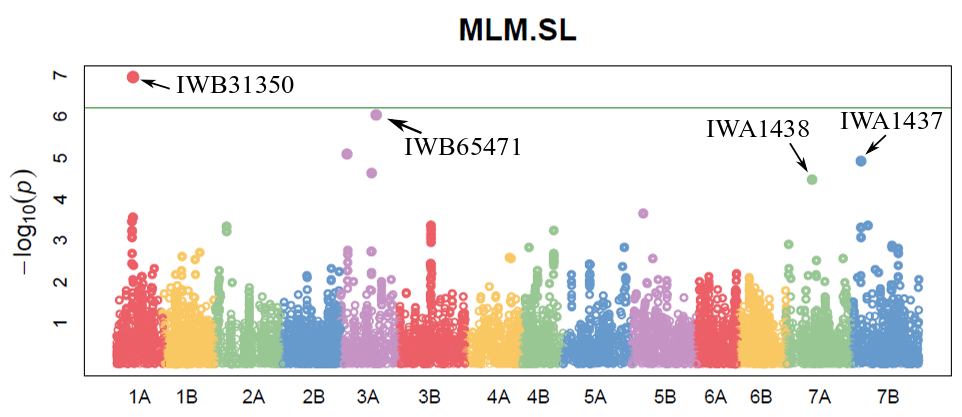** | **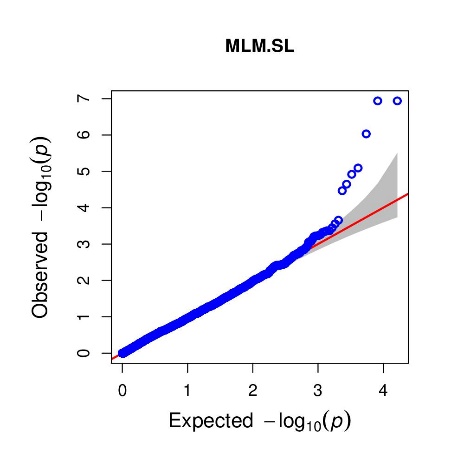** |
| --- | --- |
| A | B |

**S2 Fig 13 Spike length MTAs in SEK18 (DWV).** A) Manhattan plot. B) Q-Q plot. Vertical axes show the negative logarithm of the association *P*-value. Horizontal axes show chromosomes. Arrows indicate identified stable MTAs.

| 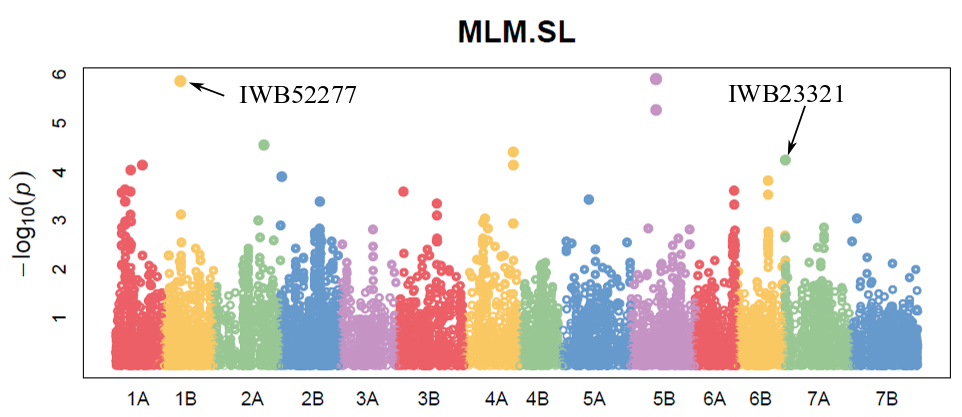 | 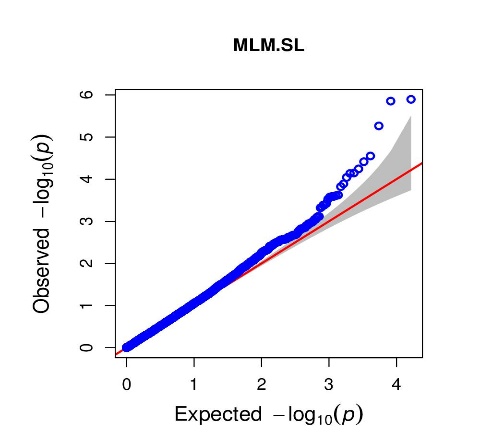 |
| --- | --- |
| A | B |

**S2 Fig 14 Spike length MTAs in SEK19 (TWC).** A) Manhattan plot. B) Q-Q plot. Vertical axes show the negative logarithm of the association *P*-value. Horizontal axes show chromosomes. Arrows indicate identified stable MTAs.

| 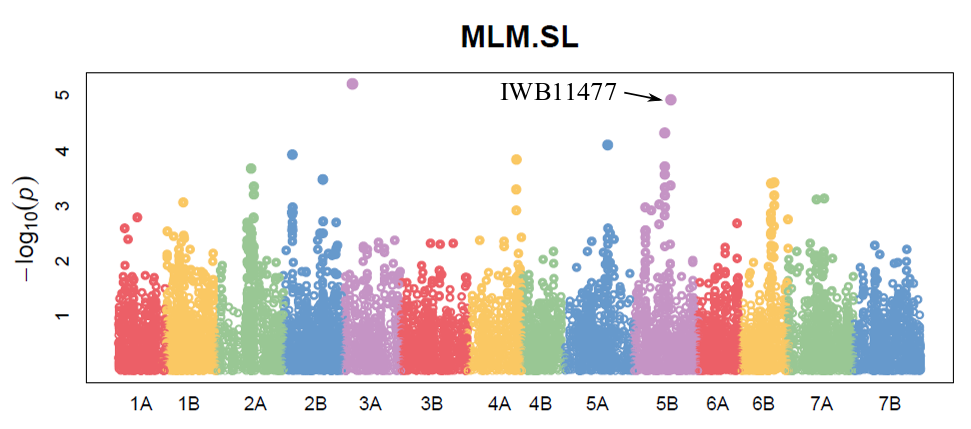 | 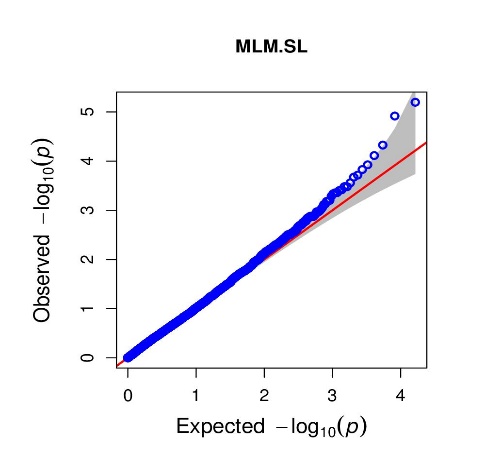 |
| --- | --- |
| A | B |

**S2 Fig 15 Spike length MTAs in NK19 (TWC).** A) Manhattan plot. B) Q-Q plot. Vertical axes show the negative logarithm of the association *P*-value. Horizontal axes show chromosomes. Arrows indicate identified stable MTAs.

| 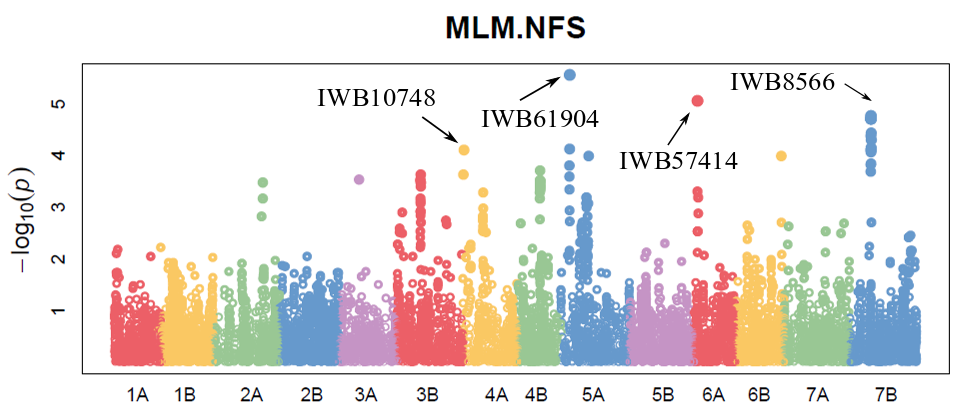 | 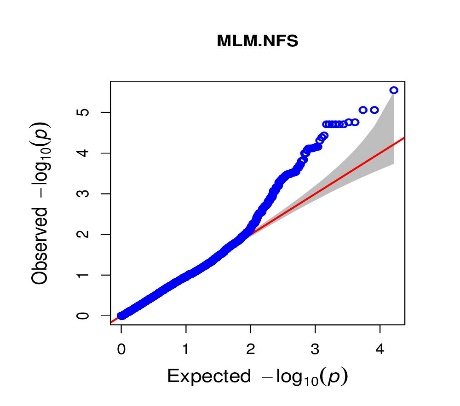 |
| --- | --- |
| A | B |

**S2 Fig 16 Number of fertile spikes MTAs in SEK18 (DWV).** A) Manhattan plot. B) Q-Q plot. Vertical axes show the negative logarithm of the association *P*-value. Horizontal axes show chromosomes. Arrows indicate identified stable MTAs.

| 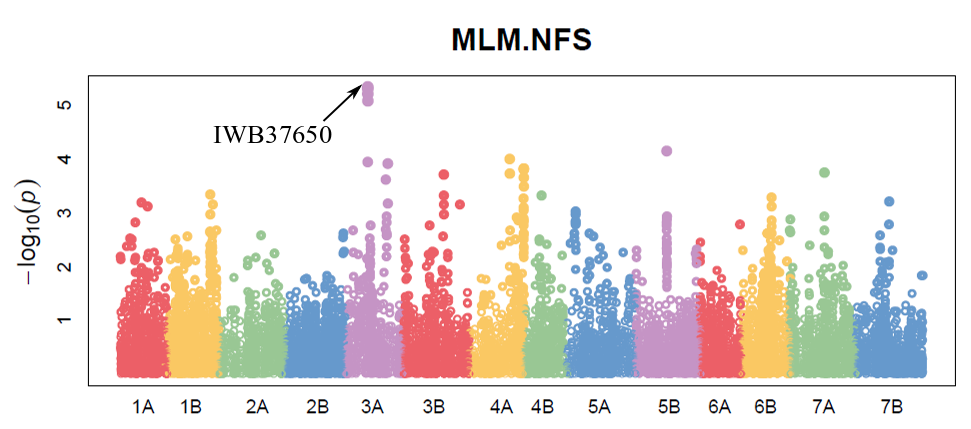 | 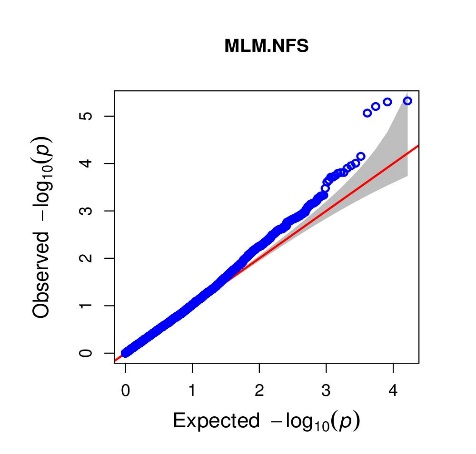 |
| --- | --- |
| A | B |

**S2 Fig 17 Number of fertile spikes MTAs in NK19 (TWC).** A) Manhattan plot. B) Q-Q plot. Vertical axes show the negative logarithm of the association *P*-value. Horizontal axes show chromosomes. Arrows indicate identified stable MTAs.

| 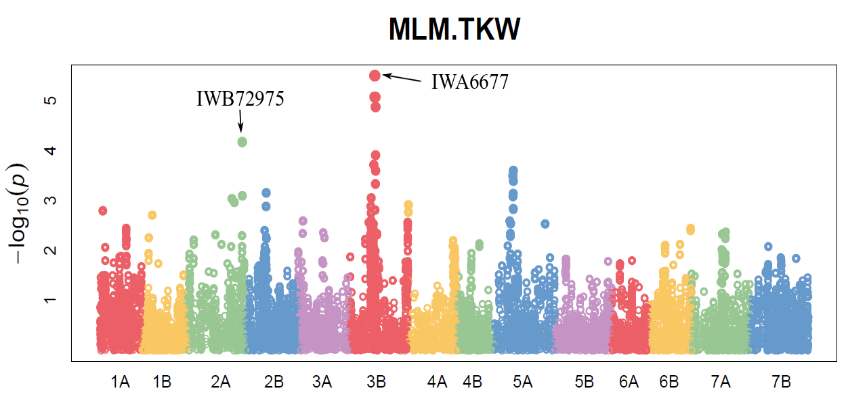 | 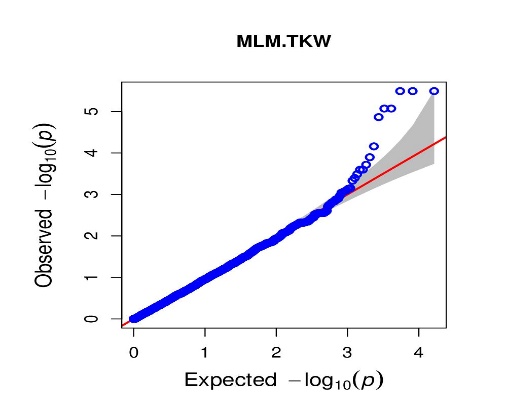 |
| --- | --- |
| A | B |

**S2 Fig 18 Thousand kernel weight MTAs in SEK18 (DWV).** A) Manhattan plot. B) Q-Q plot. Vertical axes show the negative logarithm of the association *P*-value. Horizontal axes show chromosomes. Arrows indicate identified stable MTAs.

| 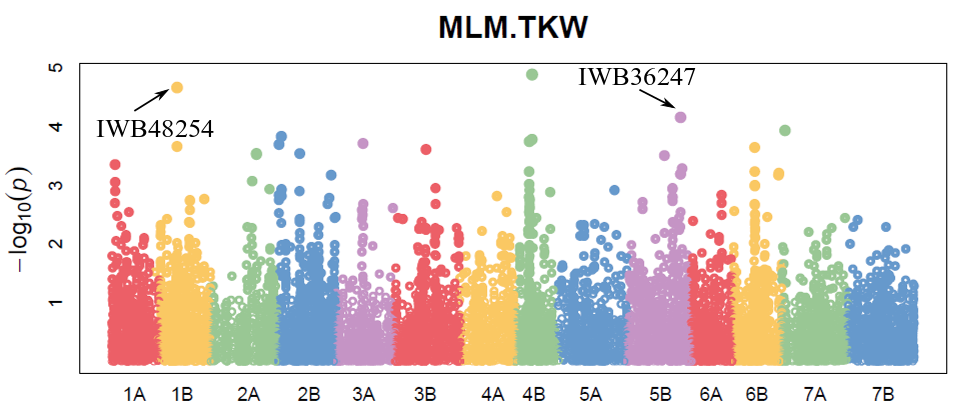 | 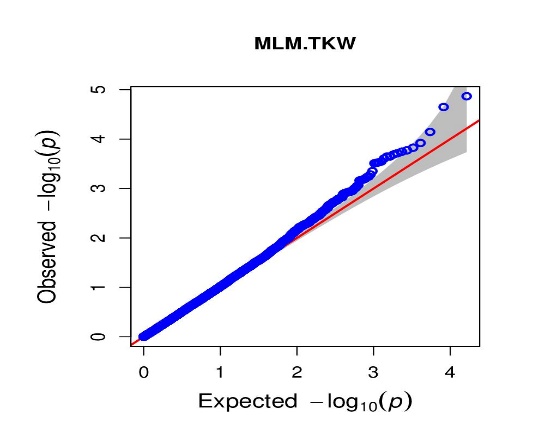 |
| --- | --- |
| A | B |

**S2 Fig 19 Thousand kernel weight MTAs in NK18 (TWC).** A) Manhattan plot. B) Q-Q plot. Vertical axes show the negative logarithm of the association *P*-value. Horizontal axes show chromosomes. Arrows indicate identified stable MTAs.

| 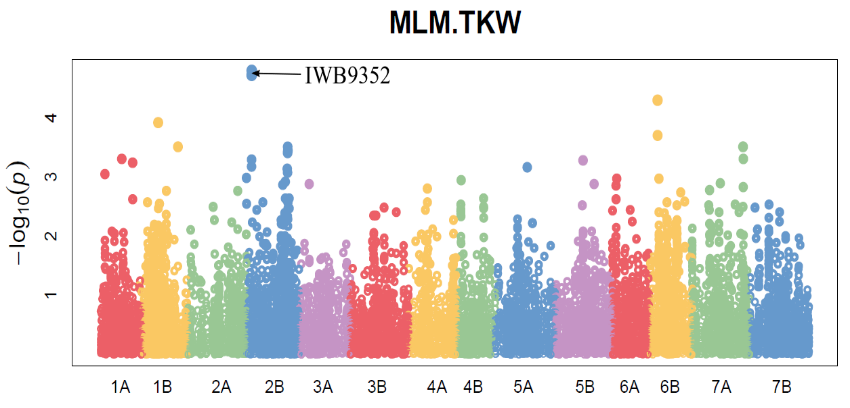 | 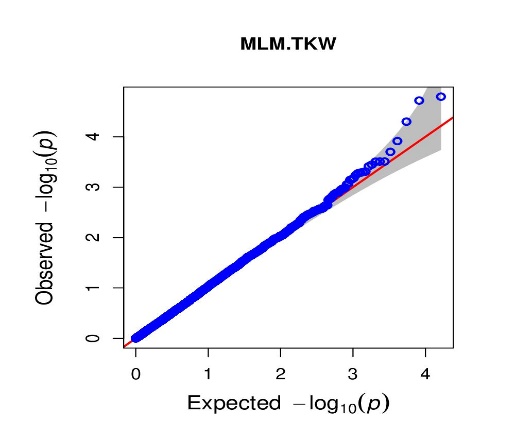 |
| --- | --- |
| A | B |

**S2 Fig 20 Thousand kernel weight MTAs in in NK19 (TWC).** A) Manhattan plot. B) Q-Q plot. Vertical axes show the negative logarithm of the association *P*-value. Horizontal axes show chromosomes. Arrows indicate identified stable MTAs.
