# Supplementary material for: Quantitative trait loci for agronomic traits in tetraploid wheat for enhancing grain yield in Kazakhstan environments
Source: PLoS One. 2020 Jun 23;15(6):e0234863. doi: 10.1371/journal.pone.0234863 (PMC7310741; doi:10.1371/journal.pone.0234863)
Supplement: S2 Table — (DOCX) [file pone.0234863.s004.docx]

| Index | South-East | | North | |
| --- | --- | --- | --- | --- |
|  | 2018 | 2019 | 2018 | 2019 |
| Veg. period Rainfall, mm | 311.6 | 396.0 | 197.5 | 197.0 |
| Veg. period, Mean *T*, °C | 20.2 | 18.7 | 17.6 | 15.7 |
| Veg. period, Max *T*, °C | 37.7 | 37.6 | 33.0 | 35 |
| Veg. period, Min *T*, °C | -1.7 | -2.9 | -2.0 | -5 |
| Soil type | Light chestnut (humus 2.0-2.5%) | | Black soil (humus 4.5-5%) | |
| Latitude | 43°21′ | | 4°10′ | |
| Longitude | 76°53′ | | 69°31′ | |
| Elevation above sea level | 740 | | 141 | |
| Date of sowing | 15 April | 28 March | 31 May | 31 May |
| Date of harvesting | 1 August | 4 August | 1 October | 12 October |
| Irrigation | Rainfed | | Rainfed | |

**Supplemental Table S2. Climate and meteorological conditions and location data at the experimental sites in two regions of Kazakhstan**
